# Mutational analysis of the pro‐peptide of a marine intracellular subtilisin protease supports its role in inhibition

**DOI:** 10.1002/prot.25528

**Published:** 2018-09-17

**Authors:** Gro E. K. Bjerga, Øivind Larsen, Hasan Arsın, Adele Williamson, Antonio García‐Moyano, Ingar Leiros, Pål Puntervoll

**Affiliations:** ^1^ Uni Research Center for Applied Biotechnology Thormøhlens gate 55, Bergen 5006 Norway; ^2^ Department of Biological Sciences University of Bergen Thormøhlens gate 53, Bergen 5006 Norway; ^3^ The Norwegian Structural Biology Centre (NorStruct), Department of Chemistry UiT The Arctic University of Norway Tromsø 9037 Norway

**Keywords:** ISP, LIPY/F‐motif, Planococcus, protease structure, subtilisin

## Abstract

Intracellular subtilisin proteases (ISPs) have important roles in protein processing during the stationary phase in bacteria. Their unregulated protein degrading activity may have adverse effects inside a cell, but little is known about their regulatory mechanism. Until now, ISPs have mostly been described from *Bacillus* species, with structural data from a single homolog. Here, we study a marine ISP originating from a phylogenetically distinct genus, *Planococcus* sp. The enzyme was successfully overexpressed in *E. coli*, and is active in presence of calcium, which is thought to have a role in minor, but essential, structural rearrangements needed for catalytic activity. The ISP operates at alkaline pH and at moderate temperatures, and has a corresponding melting temperature around 60 °C. The high‐resolution 3‐dimensional structure reported here, represents an ISP with an intact catalytic triad albeit in a configuration with an inhibitory pro‐peptide bound. The pro‐peptide is removed in other homologs, but the removal of the pro‐peptide from the *Planococcus* sp. AW02J18 ISP appears to be different, and possibly involves several steps. A first processing step is described here as the removal of 2 immediate N‐terminal residues. Furthermore, the pro‐peptide contains a conserved LIPY/F‐motif, which was found to be involved in inhibition of the catalytic activity.

## 1 INTRODUCTION

ISPs have key roles in cell cycle regulation, specifically in protein recycling by processing proteins during transition to the stationary phase.^1^, ^2^ To prevent proteolysis that may be lethal to the cell, the activity of an intracellular protease must be tightly controlled. Although a potential ISP inhibitor protein has been identified,^3^, ^4^ the primary mechanism of regulation is likely intrinsic.^5^, ^6^ In the precursor protein, an N‐terminal pro‐peptide of typically 16‐20 residues binds across the active site and inhibits activity. As shown for a few homologs,^6^, ^7^ the pro‐peptide is released by intra‐molecular maturation allowing the enzyme to act on exogenous substrates. ISPs are homodimeric,^6^ which contributes to making ISPs a structurally distinct family of subtilases. The catalytic domain of ISPs are homologous to those of other members of the Subtilisin superfamily, such as the extracellular subtilisin proteases (ESPs), which is a “Peptidase S8” domain in the Pfam classification.^8^ According to the MEROPS peptidase database,^9^ both ISPs and ESPs belong to the S08 family in clan SB.

Within this domain a catalytic triad is arranged as an aspartate, a histidine and a serine (Asp32, His64, and Ser221, respectively, referring to the processed SubtilisinE from *B. subtilis*; Uniprot ID: CAB12870). In brief, the nitrogen‐bonded protein (Nε2‐H) of His64 is hydrogen bonded to the hydroxyl group proton of Ser221. This interaction causes a charge separation of the hydroxyl, deprotonating the serine oxygen and activating it for nucleophilic attack on the carbonyl of the peptide substrate, which ultimately leads to breakage of the peptide bond.^10^ Aside from homology within the catalytic domain, significant architectural differences exist between ISPs and ESPs. The N‐termini of ESPs contain short leader sequences of about 20‐30 residues for protein secretion,^11^ followed by a pro‐domain of typically 60‐80 residues,^12^, ^13^ which is not conserved in sequence, but vital to their folding and function.^14^ In an analogous manner to the ISP pro‐peptide, the ESP pro‐domain is processed intra‐molecularly during maturation of the enzyme into an active conformation. The pro‐domain has dual roles in acting as an inhibitor,^15^, ^16^ and as a molecular chaperone that guides folding of the active enzyme.^16^, ^17^, ^18^

The first ESP structure was solved in 1969,^19^ and has since been reported for several homologues^20^, ^21^ and a number of engineered mutants.^22^ For ISPs, however, structural information is known from a single homologue, the *Bacillus clausii* ISP,^5^, ^6^ with 4 structures reported (PDB IDs: 2WVT, 2WWT, 2X8J, and 2XRM). The 4 structures represent 2 activity states: the inactive state with the inhibitory pro‐peptide bound and the active state without the pro‐peptide bound. Notably, all *B. clausii* structures are from inactive mutants carrying catalytic Ser250 to Ala mutations.

In ISPs, the leader sequence and pro‐domain of ESPs are replaced with a pro‐peptide (also termed N‐terminal extension). The pro‐peptide binds across the active site, with residues Phe4‐Leu6 forming a central β‐strand of a 3‐stranded antiparallel β‐sheet.^6^ The pro‐peptide also contains a LIPY/F motif, not found in ESPs. In *B. clausii* ISP this motif is involved in inhibiting the active site. Residues within the motif contribute to disruption of the conformation of the catalytic triad by shifting the catalytic Ser and His residues apart.^5^ According to a standardized residue nomenclature for peptide binding to the active site,^23^ residues N‐terminal to the scissile bond of the peptide substrate are termed P4, P3, P2, and P1, and those C‐terminal to the bond are termed P1’, P2’, P3’, and P4’, where the scissile bond is between P1 and P1’. The corresponding sites in the enzyme are S4, S3, S2, S1, S1’, S2’, S3’, and S4’. In *B. clausii* ISP, Leu6 and Ile7 correspond to P2 and P1 and are pointing inwards into the hydrophobic pocket at the S2 and S1 sites, respectively. Pro8 holds a unique position at the centre of a small curve, which displaces the peptide bond between Ile7 (P1 site) and Pro8 (P1’ site) out of reach of the active site Ser, whereas Tyr9 is occupying the S1’ site. The proline‐centred curve is unique in *B. clausii* ISP, and contrasts the scissile bond in ESPs, which is positioned to allow autoproteolytic processing. Altogether, the structure suggests that the residues in the pro‐peptide are involved in blocking the active site serine.^5^, ^6^

Both ESPs and *B. clausii* ISP harbor a conserved high affinity metal‐binding site occupied by a metal ion that serves a structural role.^5^, ^6^, ^24^, ^25^ The high affinity metal‐binding site in ESPs is occupied by calcium,^24^, ^26^ whereas in *B. clausii* ISP it is occupied by sodium.^5^, ^6^ In addition, *B. clausii* ISP has 2 unique binding sites for divalent metal ions, probably occupied by calcium ions, in each monomer: 1 close to the dimer interface and 1 in proximity to the active site. The latter is involved in ordering a loop that contributes to formation of 1 of the binding sites (S1) involved in catalysis. Due to the processing of the pro‐peptide and the positioning of calcium, the catalytic triad and substrate binding cleft are significantly rearranged, especially at the S1 binding site.^5^ In a proposed model for ISP regulation,^27^ it was suggested that once a minor fraction of the pool of ISPs adopts an open conformation, calcium binding takes place and reshapes the S1 binding site, which ultimately releases the pro‐peptide within this population and leads to a cascade of activation of other ISPs. The sequence of events and details of how the maturation precedes, in particular the role of calcium, are not known.

This study reports an ISP from a marine isolate, *Planococcus* sp. AW02J18, which is from a related, but phylogenetically distinct genus to *B. clausii*. Here, we present biochemical data for the recombinant enzyme, showing it is active in presence of calcium, at alkaline pH and moderate temperatures. We furthermore present a high‐resolution structure of an ISP with an intact catalytic triad and an inhibitory pro‐peptide bound across the active site. The structure supports previous findings and unique features of ISPs, such as its dimeric nature, sodium binding in the high‐affinity metal‐binding site and active site blocking by the pro‐peptide. The processing of the pro‐peptide appears however to be different from reported ISPs, possibly involving multiple processing steps. We also present mutagenesis data supporting an inhibitory role of the LIPY/F motif of the pro‐peptide.

## 2 MATERIALS AND METHODS

### 2.1 *In silico* identification of an intracellular subtilisin protease

The ISP sequence was identified from sequence‐based mining of a marine bacterial isolate, *Planococcus* sp. AW02J18 (Supporting Information Table S1). This isolate was collected during expeditions in the coastal areas of Lofoten in 2009, and is stored in a bacterial collection at the University of Tromsø. The sampling procedure and collection has been presented elsewhere.^28^ Genomic material was isolated for Illumina sequencing (MiSeq). Using a sequence‐based approach, translated genomic sequences from a marine bacterial collection were mined for subtilisin‐like proteases by searching for S08 family homologs against the MEROPS database.^9^ The ISP candidate was identified in this data set, and the sequence has been deposited in GenBank with the accession code MG786190.

### 2.2 The LIPY/F sequence conservation

Sequences homologous to *Planococcus* sp. AW02J18 ISP were identified using the UniProt blast search engine (default settings) against the UniRef90 database (UniProt release 2017_10).^29^ Sequence hit number 156, UniRef90_A0A136C445, was the first sequence to contain 2 motif mutations (LVNE) making the motif unlikely to be functional and was used to define the distance cut‐off (expect value 4e‐107; 57% sequence identity to *Planococcus* sp. AW02J18 ISP). Hence, the top 155 sequence hits were used to make a multiple sequence alignment (MAFFT, default settings).^30^ Three sequences were fragments that lacked the LIPY/F motif, and were manually removed (UniRef90: UPI00098840FB, UPI000590D2A7, UPI000689F3EC). The alignment containing the remaining 152 sequences was used to construct a sequence logo (default parameters).^31^

### 2.3 Sub‐cloning of the *isp* gene to expression vectors

To facilitate enzyme expression we used our previously developed screening procedure for subtilisin‐like serine proteases.^32^ The *Planococcus* sp. AW02J18 ISP protein sequence was used as template for gene synthesis (GenScript), and the synthetic *isp* gene was codon‐optimized to improve its expression in *E. coli*. The *isp* gene was synthesized with flanking *Sap*I sites, and delivered in a customized *Sap*I‐free pUC57 vector with kanamycin selection marker. The *isp* gene was sub‐cloned from the delivery vector to a suite of expression vectors using a fragment exchange (FX) cloning method.^33^ Construction of the expression vectors have been described previously.^32^

### 2.4 Gene truncation and mutagenesis

Truncation constructs and mutants were prepared from the pUC57 template. Primers were designed to contain a *Sap*I‐cloning site and a 15‐20 bp gene‐specific region targeting the desired truncation start. Primers in Supporting Information Table S2 were used to amplify the truncated ISP versions by PCR using Phusion polymerase. Gene fragments were purified, and cloned into the pINITIAL cloning vector by FX‐cloning.^32^ Plasmids were sequenced to confirm correct truncations. Point mutations were prepared by site‐directed mutagenesis using primers in Supporting Information Table S2. Truncation constructs and mutants were sub‐cloned into the p12 expression vector, using FX cloning.

### 2.5 Small‐scale expression and analysis of protein integrity

Small‐scale recombinant expression was carried out according to the protocol described previously^32^ in 4 mL culture volumes. Following expression, cells were collected and resuspended in 1 mL lysis buffer (50 m*M* Tris‐HCl pH 8.5, 50 m*M* NaCl, 0.25 mg/mL lysozyme, 10% (v/v) glycerol). Lysis was completed by ultrasonication using two 5‐s pulses at 40‐60% amplitude with a CV‐18 probe powered by an Ultrasonic Homogenizer 4710 (Cole Parmer). Lysates were cleared by centrifugation at 4600 × *g* for 20 min. Cleared lysate samples (representing soluble fraction) were analyzed by SDS‐PAGE and immunoblot as described previously.^32^ As background controls, lysates containing empty vector were used, herein termed GS due to the insertion of triple GS encoding sequence as a replacement of the *ccd*B gene in the expression vector.^32^

Semi‐quantitative analysis of recombinant protein in cleared extracts was performed in Image Lab 3.0 (BioRad). Target band intensities were extracted from image data of Coomassie‐stained SDS‐PAGE gels, and normalized to the total protein intensities in the lane excluding the target band intensities to adjust for variable growth rates and protein expression levels.

### 2.6 Large‐scale expression

*E. coli* MC1061 cells containing the p1:ISP, p12:ISP, or the p12:ISP‐S251A (catalytic mutant) constructs were grown in 1 L terrific broth medium (1.2% tryptone, 2.4% yeast extract, 0.4% glycerol, 17 m*M* KH_2_PO_4_, and 72 m*M* K_2_HPO_4_) supplemented with ampicillin (100 µg/mL) in 2.5 L Thomson's Ultra Yield™ flasks (Thomson Instrument Company). Protein expression was induced by 0.1% (w/v) *L*‐arabinose overnight at 20 °C with 250 rpm shaking. Cells were collected by centrifugation (JLA‐9.1000 rotor, Beckman) at 7500 × *g*, 30 min at 4 °C, and stored at −20 °C.

### 2.7 Protein purification

Frozen cell pellets from about 1 L culture were resuspended in 50 m*M* Tris HCl pH 7.5 at room temperature (RT, roughly around 20 °C), 150 m*M* NaCl and 0.25 mg/mL lysozyme. After incubation for 30 min at 37 °C and 250 rpm, the cell suspension was cooled on ice before sonication in a final concentration of 500 m*M* NaCl. Cell debris was removed by centrifugation at 20,000 × *g* for 20 min at 4 °C (JA‐25.50 rotor, Beckman). The cleared lysate was loaded onto 2 × 5 mL HisTrap FF crude columns (GE Healthcare) equilibrated with 50 m*M* Tris‐HCl pH 7.5 (at RT), 500 m*M* NaCl and 10 m*M* imidazole on the ÄKTA Pure (GE Healthcare) system. Bound proteins were eluted in the same buffer containing 800 m*M* imidazole. Fractions containing protein were pooled and dialyzed 2 times in Spectra Por® dialysis tubes (Spectrum Laboratories, Inc.) with 6‐8 kilo dalton (kDa) MWCO against 1 L 20 m*M* Tris‐HCl pH 7.5 overnight at 4 °C. 1 m*M* CaCl_2_ was added to a 50 µg/mL ISP solution and incubated overnight at RT during slow stirring, yielding what we herein term Asn3‐ISP (approx. 35 kDa). Protein solutions were concentrated using Amicon 10 kDa MWCO spin‐filter columns (Merck) with buffer exchange to 50 m*M* Tris HCl pH 7.5, 50 m*M* NaCl and stored in aliquots at 4 °C at concentrations 80 mg/mL (WT) and 150 mg/mL (mutant). From 1 L expression culture yields of 0.2 g of purified Asn3‐ISP (no tags), and 0.4 g of the catalytic mutant (with C‐terminal His‐tag) were typically achieved. Purity and protein mass estimation was assessed by quantitative analysis in Image Lab 3.0 (BioRad), by extracting the target band intensities from image data of Coomassie‐stained SDS‐PAGE gels.

The size exclusion chromatography experiments were ran using a pre‐calibrated Superdex 200 10/300 GL (GE Healthcare) column on the ÄKTA Explorer (GE Healthcare) system. The system was equilibrated using the loading buffer (50 m*M* Tris‐HCl pH 7.5, 150 m*M* NaCl). 500 µl Asn3‐ISP at 0.8 mg/mL concentration was loaded onto the column in loading buffer in the absence or presence of 2 m*M* CaCl_2_ or 1 m*M* EDTA. The CaCl_2_ supplemented sample was prepared immediately before the chromatography experiment to avoid autolysis, and the EDTA‐treated samples were prepared overnight in order to allow time for chelation.

Mass spectrometry (MS) analyses were performed at the PROBE facility (University of Bergen, Norway). N‐Terminal amino acid sequencing was carried out at Alta Bioscience (University of Birmingham, United Kingdom).

### 2.8 Protease activity assays

The protease fluorescent detection kit (Sigma‐Aldrich) was used for routine detection of proteolytic activity as previously described.^32^, ^34^ Briefly, 10 µL lysate or 5 μ*M* enzyme was assessed for activity on FITC‐casein in 50 m*M* TrisHCl pH 8.5 (at RT), 50 m*M* NaCl, in absence or presence of 1 m*M* CaCl_2_ in a total volume of 50 μL at 37 °C for 1 h unless otherwise stated. Temperature optimum was assayed using the FITC‐casein assay. For the mutants, activity was assessed using EnzChek™ Protease Assay Kit (ThermoFischer). 10 μg/mL BODIPY FL casein was prepared by resuspending the substrate in 50 m*M* Tris‐HCl pH 8.5 (at RT) and 50 m*M* NaCl. 12.5 μL of BODIPY‐FL casein was used per reaction, with 10 μL cleared extract in 50 m*M* Tris pH 8.5 (at RT), 50 m*M* NaCl and 1 m*M* CaCl_2_ in a final volume of 100 μL. Samples were incubated at 37 °C for 1 h, and fluorescence was read.

pH optimum was determined using 1 µ*M* Asn3‐ISP, 350 µ*M* N‐succinyl‐AAPF p‐nitroanilide (Sigma‐Aldrich) in 50 m*M* NaCl, 1 m*M* CaCl_2_, and 50 m*M* buffer (citrate buffer pH 3.0‐6.0, acetate buffer pH 4.0‐6.0, sodium phosphate buffer pH 6.0‐8.0, Tris‐HCl buffer pH 7.0‐9.0 and glycine buffer pH 9.0‐11.0). Reaction was run at 25 °C for 20 min, in presence of excess substrate.

### 2.9 Determining the specific activity

Specific activity was determined using a protease colorimetric detection kit (Sigma‐Aldrich). To avoid assay interference with amino groups from Tris, Asn3‐ISP was dialyzed against 25 m*M* borate/NaOH pH 8.2, 50 m*M* NaCl before assaying. Casein was solubilized in water at pH 8.3. One unit is defined as the amount of enzyme that will hydrolyze casein to produce color (as determined by addition of Folin‐Ciocalteu's Reagent) equivalent to 1.0 µmole tyrosine per minute at pH 8.3 at 37 °C in presence of 10 m*M* CaCl_2_.

### 2.10 Differential scanning calorimetry

Prior to Differential Scanning Calorimetry (DSC) measurements, aliquots of Asn3‐ISP at approximately 1 mg/mL were dialyzed into the following conditions overnight at 4 °C: 50 m*M* Hepes pH 8.0, 50 m*M* NaCl (DSC buffer); DSC buffer with 2 m*M* CaCl_2_; DSC buffer with 1 m*M* ethylenediaminetetraacetic acid (EDTA). Thermal unfolding transitions were measured using a Nano‐Differential scanning CalorimeterIII (Calorimetry Sciences Corporation) from 5 to 75 °C with scan rates of 1 °C/s. Buffer from the final dialysis step was used as a reference. Data were analyzed using the NanoAnalyze software (TA Instruments).

### 2.11 Crystallization

Crystallization experiments were performed with a stock solution of purified Asn3‐ISP at 30 mg/mL in 50 m*M* TrisHCl pH 7.5 (at RT), 50 m*M* NaCl. Initial crystallization conditions were screened using the vapor diffusion sitting drop method set up by a Phoenix crystallization robot (Art Robbins Instruments). The plates were set up with 60 µl reservoirs solutions and sitting drops with equal amounts of reservoir solution mixed with protein stock solution in a total drop volume of 1 µl. The screens were incubated at 20 °C. Diffraction‐quality crystals were obtained from 6 conditions, as outlined in Supporting Information Table S3.

### 2.12 X‐ray data collection

Crystals grown in 0.25 M NH_4_Ac, 22% PEG 1500, 0.1 M Na‐Citrate pH 4.0, were transferred through a cryoprotectant solution (crystallization conditions with 20% (v/v) glycerol added, thereafter mounted in a nylon loop and flash‐cooled in liquid N_2_. X‐ray diffraction data were collected at the European Synchrotron Radiation Facility (ESRF; Grenoble, France) beamline ID23EH1. The data were integrated by XDS/XSCALE,^35^ scaled and analyzed by programs in the CCP4 program suite^36^ through autoPROC.^37^ A summary of the data collection statistics is found in Table 1.

**Table 1:** Data collection and processing statistics^a^

Diffraction source/Beamline	ESRF ID23EH1
Wavelength (Å)	0.98
Detector	Q315R CCD (ADSC)
Crystal‐to‐detector distance (mm)	214.77
Rotation range pr. image (°)	0.1
Total rotation range (°)	130
Space group	*P*2_1_2_1_2_1_
a, b, c (Å)	70.17, 85.18, 104.58
α, β, γ (°)	90, 90, 90
Mosaicity (°)	0.2
Resolution range (Å)	66.05‐1.30 (1.32‐1.30)
Total No. of reflections	675238 (36457)
No. of unique reflections	142753 (7606)
Completeness (%)	92.2 (99.1)
Multiplicity	4.7 (4.8)
<I/σ (I)>	16.4 (2.2)
*R*_p.i.m._	0.026 (0.412)
Wilson B‐factor (Å^2^)	17.39

a Values in parentheses are for the outermost shell

### 2.13 Structure determination

The crystal structure was solved by molecular replacement using MolRep in the CCP4 program package^36^ with 2XRM^5^ as search model (a representative structure of the homologous ISP from *B. clausii*). The initial refinement was executed in Refmac^38^ followed by automated model improvement in Buccaneer.^39^ The manual building was done in Coot^40^ interspersed by cycles of refinement in Phenix^41^ and resulted in final *R*_cryst_/*R*_free_ values of 13.04/15.03. A summary of the refinement statistics is shown in Table 2. The atomic coordinates and structure factors have been deposited in the RCSB Protein Data Bank (http://www.rcsb.org) with the accession code 6F9M. Figures presented in the results section were generated using Chimera.^42^

**Table 2:** Structure determination and refinement statistics

Resolution range (Å)	44.563‐1.298
Completeness (%)	92.19
No. of reflection, working set	142740
No. of reflection, reference set	1992
Final *R*_cryst_	13.04
Final *R*_free_	15.03
MolProbity score	1.315
Clashscore	3.35
No. of non‐H atoms	
Protein^a^	4548
Water	549
Other^b^	30
Total	5127
R.m.s. deviations	
Bonds (Å)	0.007
Angles (°)	0.962
Average B factors (Å^2^)	
Overall	23.57
Protein	21.87
Water	37.20
Other*	32.01
Ramachandran plot (%)	
Preferred	96.87
Allowed	2.61
Outliers	0.52

a Two molecules pr. asymmetric unit.

b Two molecules of acetate, sodium and triethylene glycol (Peg 3) were identified in the electron density and modeled. These occupy similar positions around the 2 protein molecules in the asymmetric unit.

## 3 RESULTS

### 3.1 A new intracellular subtilisin protease with a conserved LIPY/F motif

**Figure 1:**
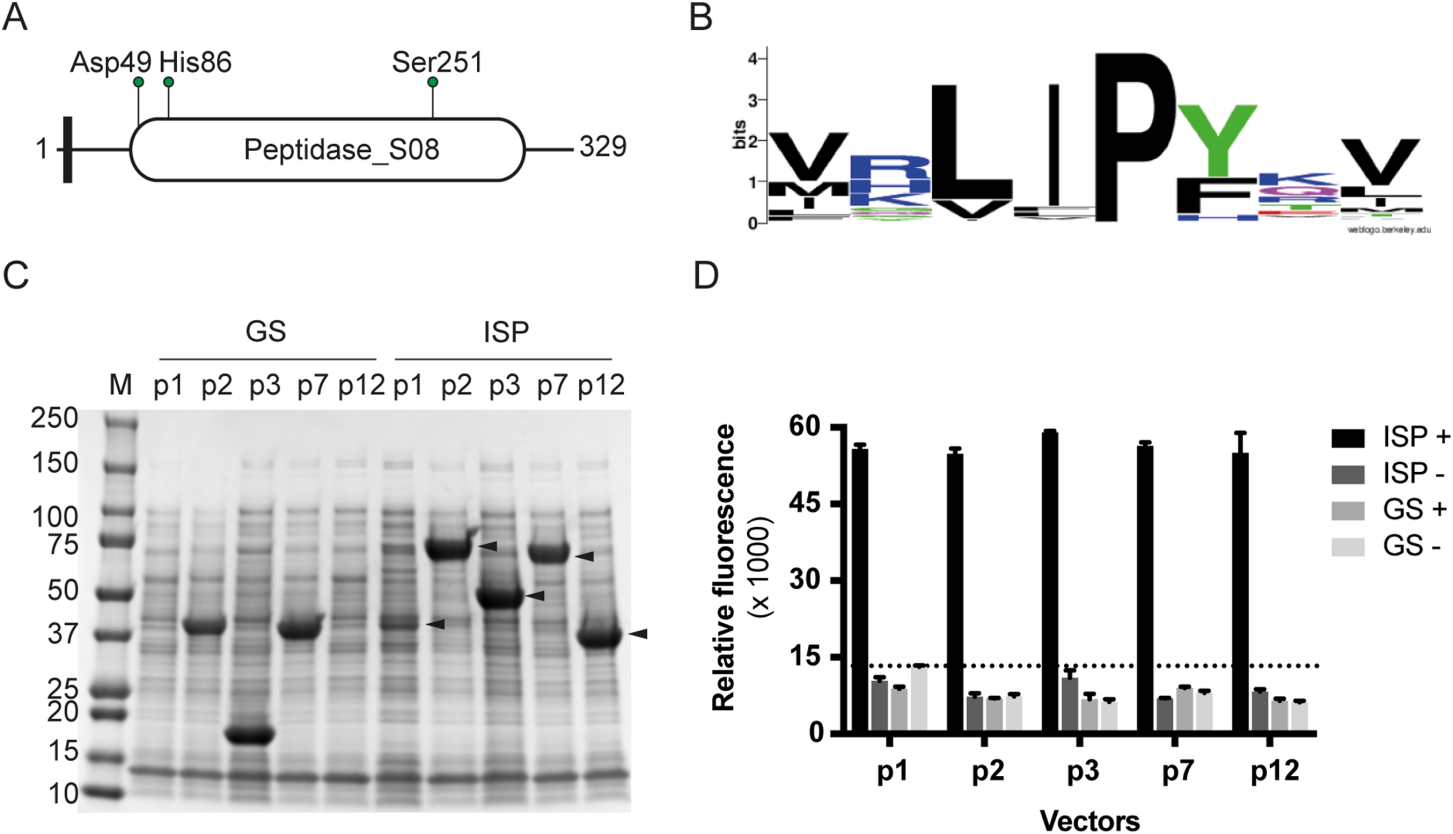
Overexpression and activity assessment of the recombinant ISP. A, A cartoon of the ISP architecture drawn to scale. Black box, LIPY/F motif; oval circle, Peptidase_S08 Pfam domain (PF00082); green pins point to residues involved in catalysis (catalytic triad). B, Sequence logo showing the evolutionary conservation of the LIPY/F motif based on an alignment with 152 ISP sequences. C, ISP constructs were produced from multiple vectors, and cleared lysates were inspected on SDS‐PAGE for the presence of soluble overexpressed proteins. Arrows indicate soluble ISP proteins. Fusion partners from the various vectors are: p1, N‐terminal His‐tag; p2, N‐terminal His‐tag and MBP; p3; N‐terminal His‐tag and SUMO protein; p7, N‐terminal MBP and C‐terminal His‐tag; p12; C‐terminal His‐tag. Empty vector controls (GS) will produce fusion partners only, wherein MBP and SUMO can be observed on SDS‐PAGE. M, BioRad's Precision Plus Protein™ Dual Color Standard. D, Cleared lysates (see C for details), including empty vector controls (GS) were assayed over night with FITC‐casein, in the presence (+) or absence (−) of 1 m*M* CaCl_2_. Dotted line indicates the highest data point for background measurements (in the absence of calcium)

A previously uncharacterized protease from *Planococcus* sp. AW02J18 was identified in an enzyme discovery initiative as a candidate for expression in *E. coli* (Supporting Information Table S1). According to sequence analysis, this protease contained a catalytic domain (Peptidase_S8/PF00082) as annotated by Pfam (residues 40‐311, Figure 1A). Sequence analysis also revealed that it shared 53% sequence identity to the previously described intracellular subtilisin protease (ISP) from *B. clausii* 6 (Supporting Information Figure S1). As expected from SignalP analysis, the ISP sequence does not contain a leader sequence to direct its export,^43^ and is thus predicted to have an intracellular localization. Instead, the *Planococcus* sp. AW02J18 ISP contains a short pro‐peptide with a LIPY‐sequence at the N‐terminus, also identified in other homologues (Supporting Information Figure S1). Although the LIPY sequence has been reported as a conserved motif,^6^ evidence of its conservation has not previously been presented. To analyze the evolutionary conservation of the motif, sequences homologous to the *Planococcus* sp. AW02J18 ISP were collected. Using 152 UniRef90 sequences in a sequence alignment, we analyzed conservation of the motif in a context with 2 flanking residues on each side (8 residue window). A LIPY/F motif is derived from the alignment (Figure 1B). A hydrophobic leucine or valine, or in rare cases an isoleucine occurs at the first position. At the second position, the motif contains most often a hydrophobic isoleucine, but in certain sequences phenylalanine, leucine or valine. The third position is occupied by a highly conserved proline found in all but 2 sequences. This residue is structurally significant as part of the proline‐centred curve in *B. clausii* ISP, which positions the scissile bond between proline and the previous residue out of reach for autocatalysis. At the fourth position, an aromatic tyrosine, phenylalanine or in rare cases histidine occurs. At flanking positions of these 4 residues some consensus occurs, such as a charged residues at proximate positions to the LIPY/F motif, and hydrophobic residues at positions 2 residues upstream and downstream (Figure 1B). A 4‐residue motif can be expressed using the Prosite pattern syntax as [LVI]‐[IFLV]‐P‐[YFH].

### 3.2 The first 2 residues of the calcium‐dependent ISP is processed

**Figure 2:**
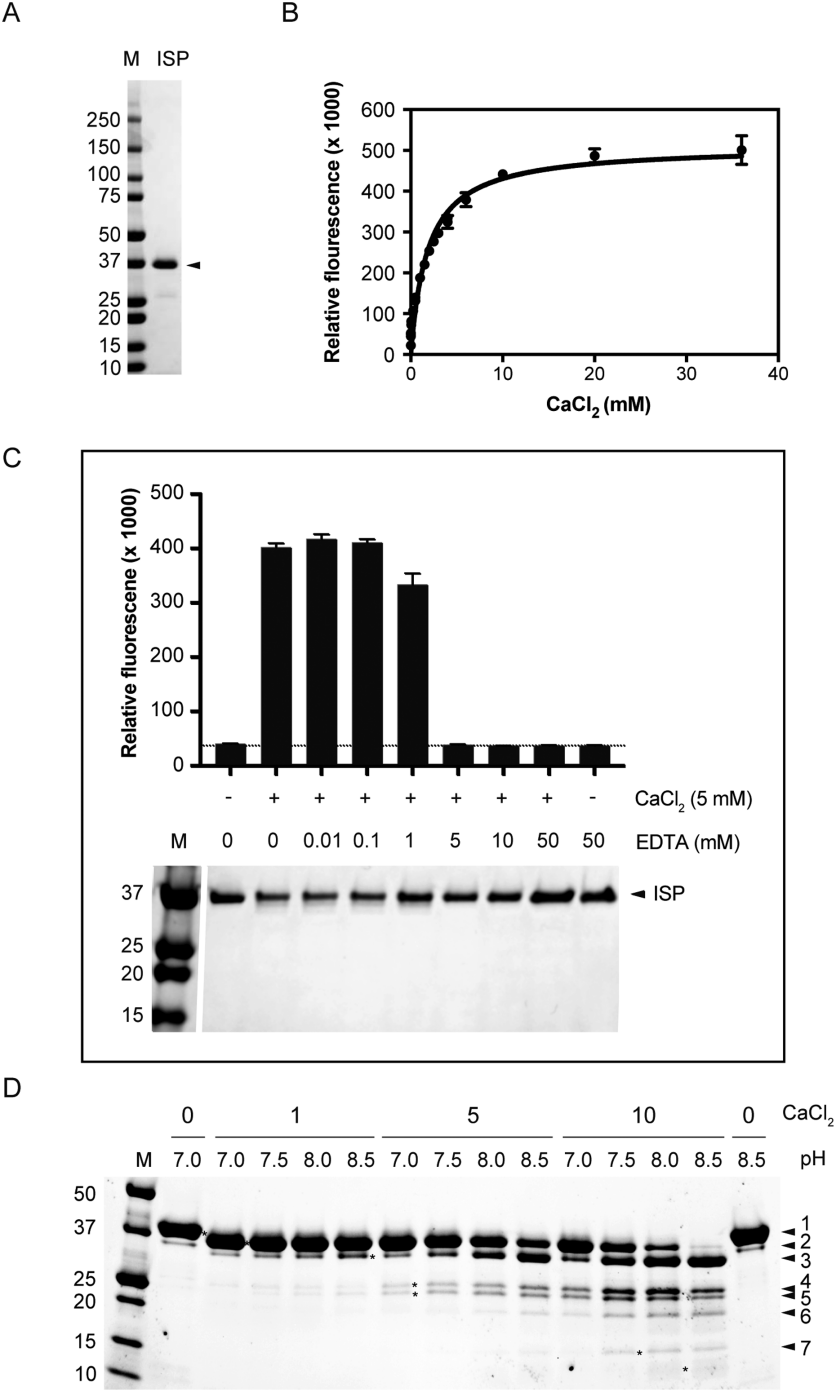
ISP activity, stability and processing. A, SDS‐PAGE analysis of purified Asn3‐ISP. Purity was estimated to 95% by inspection of lane intensity. B, Using the FITC‐casein assay, 4 µ*M* Asn3‐ISP was incubated with increasing concentration of CaCl_2_ at 37 °C for 1 h. 50% activity is achieved with 2.5 m*M* CaCl_2_. C, The activity of 1 μg Asn3‐ISP (as in A) was measured in the presence or absence of 5 m*M* CaCl_2_ and concentrations of EDTA up to 50 m*M* (upper panel). Dotted line represents the average of buffer (37248 units) in presence of 5 m*M* CaCl_2_ and 5 m*M* EDTA. Lower panel shows SDS‐PAGE containing 0.5 μg Asn3‐ISP treated with CaCl_2_ and EDTA as in the activity assay. M, BioRad's Precision Plus Protein™ Dual Color Standard. D, The His‐ISP protein construct (p1‐construct, numbered 1) was used to investigate calcium‐induced maturation. 1, 5 or 10 m*M* CaCl_2_ was added to 2 μg enzyme at pH range 7.0‐8.5 at room temperature for incubation overnight before analysis on SDS‐PAGE. Theoretical mass of Asn3‐ISP (numbered 2), 35 kDa. M, BioRad's Precision Plus Protein™ Dual Color Standard

The full‐length *isp* gene from *Planococcus* sp. AW02J18 was sub‐cloned to a suite of expression vectors for heterologous expression. From SDS‐PAGE analysis, we found that all recombinant constructs yielded soluble enzyme, but that solubility was further improved by use of fusion tags (Figure 1C). Since many serine proteases require calcium for proper folding and structural stability, activity was assessed on fluorescein isothiocyanate (FITC) conjugated casein in the absence or presence of calcium ions. Compared to extracts from strains carrying empty vectors, all recombinant enzymes were active, but required calcium for activity (Figure 1D). The p1‐construct encoding an N‐terminal deca‐histidine (His) tag was chosen for in‐depth characterization due to its potential to yield a recombinant enzyme that would mimic the native processed ISP, and ease downstream purification (Figure 1). In the absence of calcium, immobilized metal affinity chromatography (IMAC) was used for protein purification of His‐ISP (approx. 38 kDa). In analogy to the ISP from *B. clausii*, the enzyme was incubated in presence of calcium to mature by autoproteolysis. From SDS‐PAGE we obtained a “matured ISP”, with an expected lower mass (∼35 kDa) than full‐length, of 95% purity (Figure 2A). With N‐terminal sequencing we determined the starting residue on this protein entity to Asn3; we thus termed this protein Asn3‐ISP. Using Asn3‐ISP, we found that increasing concentrations of calcium had a positive effect on activity (Figure 2B), whereas EDTA inactivated the enzyme (Figure 2C). From SDS‐PAGE analysis of the reaction products, we found that Asn3‐ISP was further processed or degraded in presence of calcium (Figure 2C). In absence of calcium or in calcium‐depleted reactions, the enzyme was however persistent against proteolysis (Figure 2C), and could be stored for 1 month without any effect on activity (Supporting Information Figure S2).

To further understand the processing, calcium chloride was added at various concentrations to the full‐length recombinant enzyme (His‐ISP) at a pH range 7.0‐8.5. SDS‐PAGE revealed that 2 processed ISP species < 37 kDa appeared in presence of 1 m*M* CaCl_2_ (Figure 2D, protein bands numbered 2‐3). N‐terminal sequencing was performed on these 2 processed protein species, but data were only conclusive for the uppermost processed protein. In this protein, the artificial N‐terminal residues (His‐tag and 3C protease site) and the 2 first native residues of the ISP (residues Met1, Lys2) were processed. This confirms what is referred to as the Asn3‐ISP. Increasing the concentration of calcium chloride up to 10 m*M* led to further processing as well as the appearance of degradation products (that is, fragments smaller than the 31 kDa peptidase domain). MS analyses were performed on various entities after calcium‐induced activation, with identification of ISP peptides in all samples (Supporting Information Figure S3 and Table S5). No obvious sequential pattern between protein entities was identified. Tag‐removal was confirmed by immunoblot analysis and compared to a catalytic mutant designed by replacing the catalytic Ser251 with Ala (Supporting Information Figure S4). The processing of the recombinant ISP from *Planococcus* sp. AW02J18 appears to occur in multiple steps.

### 3.3 *Planococcus* sp. AW02J18 ISP operates at moderate temperatures and alkaline pH

**Figure 3:**
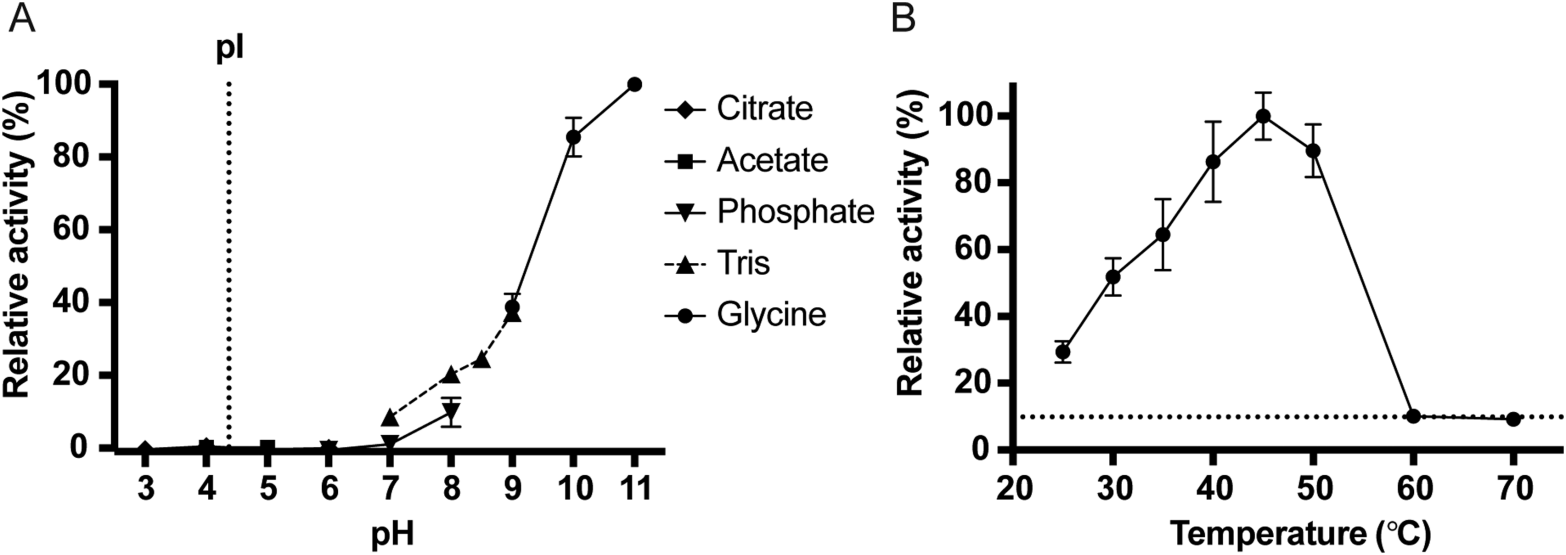
pH and temperature optimum of ISP. A, Using the N‐succinyl‐AAPF p‐nitroanilide peptide, the activity of 1 μ*M* Asn3‐ISP at pH 3.0‐11.0 was measured in the initial rate of the reaction at 25 °C. Background from buffer was subtracted and data was made relative to measurement data at pH 11.0. Citrate buffer was used for pH 3.0‐6.0 (diamonds), acetate buffer for pH 4.0‐6.0 (square), sodium phosphate buffer from pH 6.0‐8.0 (down‐pointing triangles), Tris‐HCl buffer for pH 7.0‐9.0 (up‐pointing triangles, dotted line between points) and glycine buffer (circles) for pH 9.0‐11.0. Error bars represent deviation between 2 replicas in 1 representative experiment. The pI of the ISP is estimated to approximately 4.4 (vertical dotted line). B, Activity of 5 μ*M* Asn3‐ISP was monitored in the FITC‐casein assay across a temperature range of 25‐70 °C. Background was subtracted and made relative to the measured data at 45 °C. CaCl_2_ was added immediately before assaying. The assay took place for 1 h at the respective temperatures. Error bars represent deviation between data points from 3 independent experiments. The horizontal dotted line represents the highest background measurement

To identify its optimal conditions for further activity assessments, Asn3‐ISP was characterized with respect to the specific activity, temperature and pH optimum in casein assays (Figure 3). It was found to operate optimally at pH 11.0, but was active across pH 7.0‐11.0, whereas no activity was observed below pH 6.0 (Figure 3A). Precipitation was observed at pH 4.0 in both citrate and acetate buffers, likely explained by an estimated pI around 4. The temperature optimum was found to be around 45 °C (Figure 3B). No activity was identified above 60 °C, which indicates that the protein is destabilized at high temperatures. Using optimal temperature (45 °C) in alkaline conditions (pH 8.3) and 10 m*M* CaCl_2_ the specific activity of the ISP was determined on casein to be 13 ± 1 U/mg.

**Figure 4:**
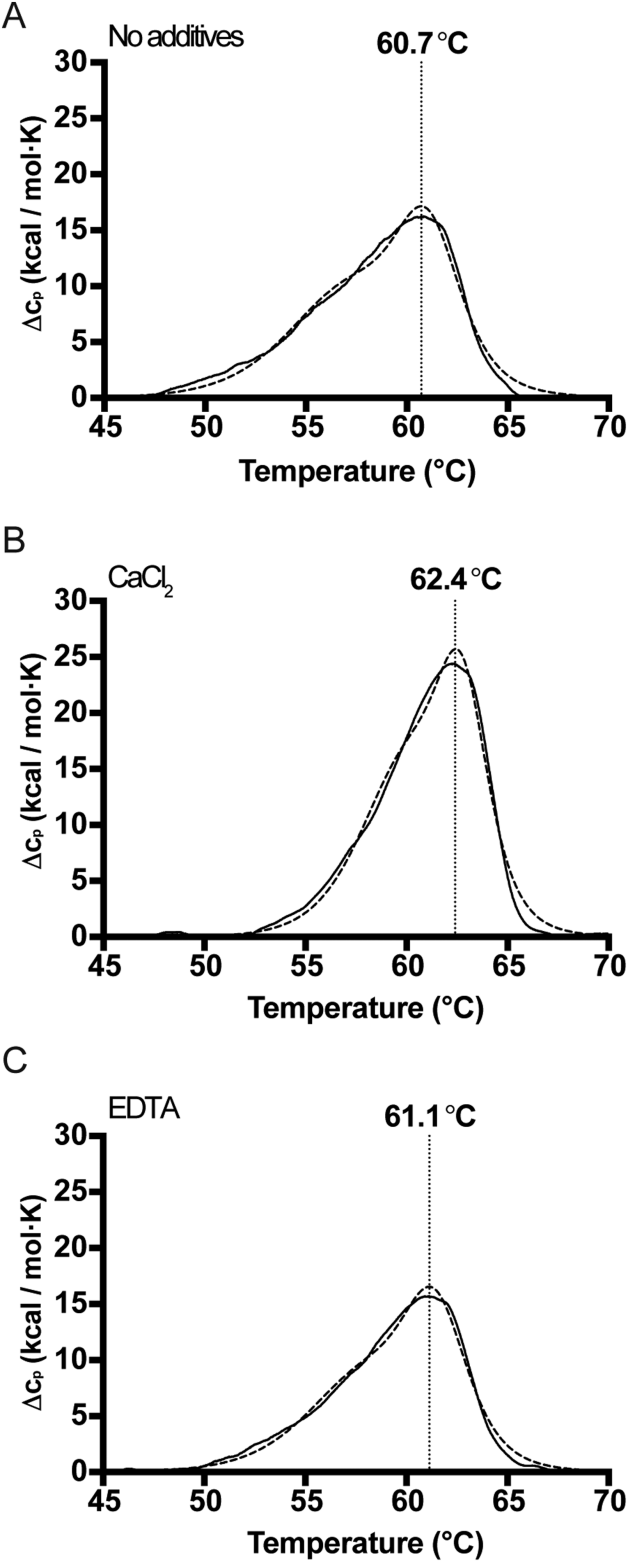
Thermal unfolding transitions of Asn3‐ISP. Unfolding was measured in metal‐depleted ISP in 3 conditions; A, without additives, B, in presence of 2 m*M* CaCl_2_, and C, in presence of 1 m*M* EDTA. Representative thermograms are shown after subtraction of buffer scans and fitting of a sigmoidal baseline (solid lines). The sum of the 2 two‐state models fitted to each thermogram is shown with dashed lines. The Tm of the higher temperature transition is indicated with a vertical dotted drop‐line for comparison

To determine the thermal unfolding temperature of Asn3‐ISP, DSC measurements were carried out (Figure 4). The enzyme unfolded as a single peak, which could be fitted to 2 two‐state transitions with melting temperatures (Tm) separated by approximately 3.0 °C (Table 3). In the DSC data, the apparent Tm in absence of calcium and EDTA was around 60 °C, which is consistent with the data on temperature optimum and stability (Figure 4A). Addition of CaCl_2_ increased the directly measured *T*_max_ by 1.7 °C, and the apparent Tm by up to 3.0 °C indicating that calcium has a stabilizing effect on the enzyme (Figure 4B). The presence of EDTA slightly increased the apparent *T*_m_ (Figure 4C). Repeat scanning did not give rise to any subsequent unfolding transitions, indicating that ISP does not refold on the timescale used for this experiment; therefore the thermodynamics of unfolding were not analyzed further. No exothermic signals indicative of aggregation were present in the raw data (not shown), and no visible precipitate was observed suggesting that these data can be used in a comparative manner to understand the effect of EDTA and calcium on the system.

**Table 3:** Thermal denaturation measured by DSC^a^

Treatment	Δ*H*_cal_	*T*_max_	Δ*S*	Δ*H*_vH_1	*T*_m1_	Δ*H*_vH_2	*T*_m2_	Δ*D*
None	120.1	60.7	0.356	105.0	57.2	209.1	61.0	0.49
2 mM CaCl_2_	145.1	62.4	0.421	142.0	60.1	266.0	62.8	0.70
EDTA	125.0	61.1	0.345	111.7	58.0	215.2	61.4	0.39

a Δ*H*_cal_ (calorimetric enthalpy), Δ*S* (entropy of unfolding) and *T*_max_ are calculated directly from the unfolding transition. Δ*H*_vH_ and *T*_m_ are derived from fitting 2 two‐stated scaled models to each transition after subtraction of buffer scans and a sigmoidal baseline

### 3.4 Structure of ISP with an intact catalytic triad and pro‐peptide

**Figure 5:**
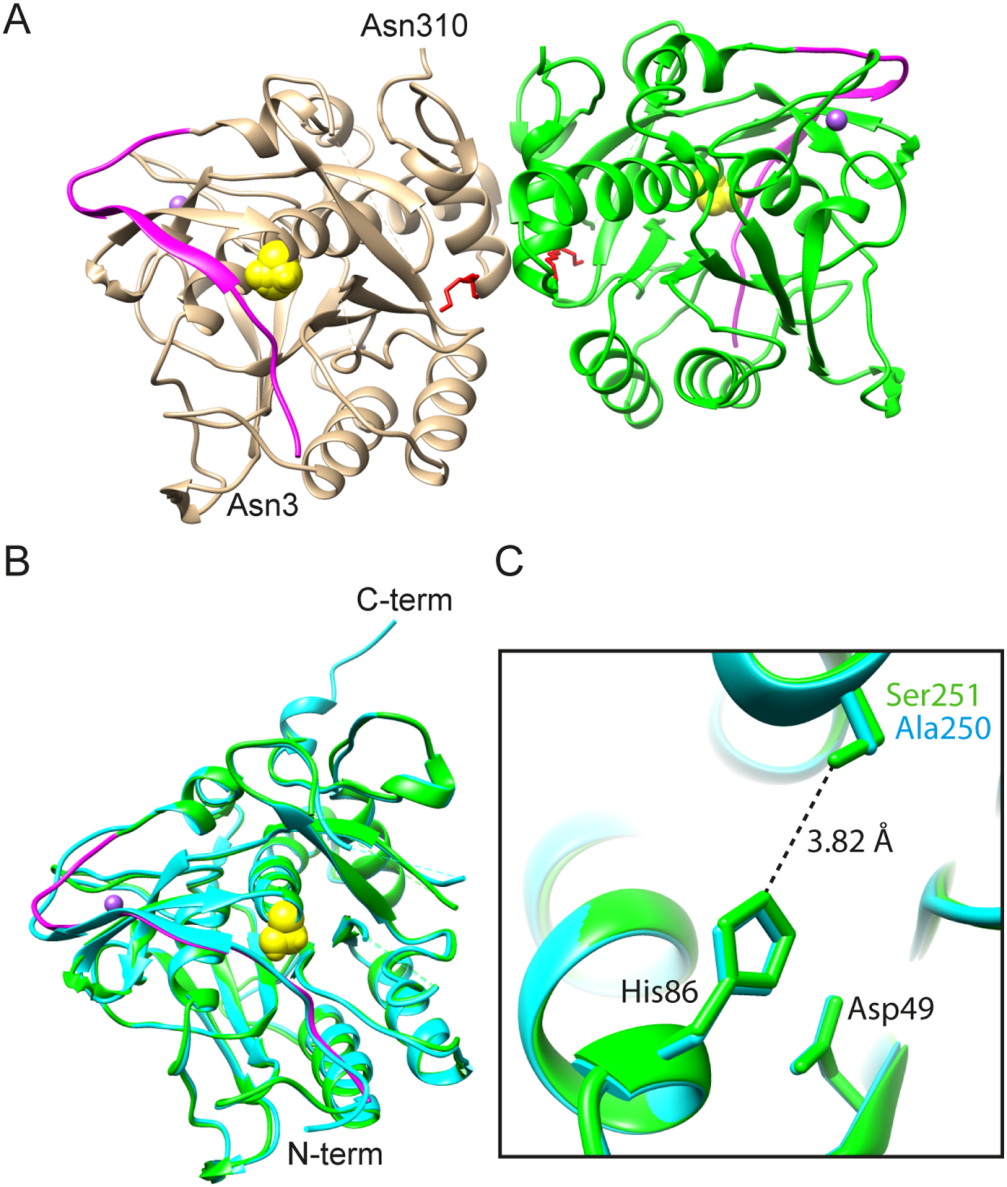
Structure of *Planococcus* sp. AW02J18 ISP. A, Dimer presented in ribbon. The pro‐peptide (residues 3‐20) is shown in magenta in both monomers (chain A in tan, chain B in green). The N‐terminal and C‐terminal residues are labelled in monomer A. The catalytic Ser251 is shown as a yellow sphere. The Peg molecules in the dimer interface are shown in red. B, Superposition of the ISP from *Planococcus* sp. AW02J18 (green, chain B) on the ISP template from *B. clausii* (blue, PDB ID: 2X8J, chain A). Ser251 in ISP from *Planococcus* sp. AW02J18 is shown as a yellow sphere, and its pro‐peptide (residues 3‐20) is shown in magenta. C, The catalytic triad of *Planococcus* sp. AW02J18 ISP (green, chain B) and the catalytic mutant of *B. clausii* ISP (cyan, chain A). Distance (Å) between Ser251 and His86 in *Planococcus* sp. AW02J18 ISP is given as a dashed line

ISPs are distinct from ESPs with regards to the N‐terminal pro‐peptide, their dimeric structure, and the sodium binding in the high affinity metal binding site,^5^, ^6^ but details regarding their maturation are still unclear. To shed light on the latter, the crystal structure of the Asn3‐ISP was determined by X‐ray crystallography to a resolution of 1.3 Å (Figure 5). In addition to being the second unique structure of an ISP, it is the first structure of an ISP with a native catalytic triad, and it represents the highest resolution structure of this enzyme family to date. The structure of the ISP (residue 3‐310) is dimeric, with each monomer including an almost intact pro‐peptide bound across the active site. Using size exclusion chromatography (SEC) the molecular weight of Asn3‐ISP in solution was estimated. The absence of calcium and presence of EDTA gave similar elution profiles in SEC and mass estimates; here, 13.4 mL and 115 kDa, respectively (Supporting Information Figure S5). In presence of calcium, the Asn3‐ISP eluted in 2 peaks at 13.2 and 14.1 mL corresponding to masses of approximately 128 and 88 kDa, respectively (referring to the regression line produced from known calibration proteins). Based on the theoretical mass of Asn3‐ISP being 35 kDa, this suggests 3.3 monomers per oligomer in absence of calcium. In presence of calcium the oligomeric state shifts to a mixed population of both 3.7 and 2.5 monomers per oligomer. In the structure, there are 2 molecules of triethylene glycol (Peg3) symmetrically bound at the dimer interface distant from the active site (Figure 5A), which may be adducts of Peg 1500 during crystallization or introduced during recombinant expression. In 3 structures of ISP from *B. clausii*, similar molecules are bound in this region: a strontium ion and a tetraethylene glycol molecule bound in an overlapping position (PDB ID: 2XRM); 3 water molecules bound in the same region (PDB ID: 2WWT); and Peg3 (PDB ID: 2X8J) almost perfectly overlapping the conformation observed in the *Planococcus* sp. AW02J18 ISP structure.

The structure contains a catalytic core (residues 20‐310) overlapping the Pfam assigned Peptidase_S8 domain (residues 40‐311). The first 2 residues, 2 loop regions (residues 184‐191 and 217‐223), and the C‐terminal 20 residues are not defined in the electron density. Superpositioning of *Planococcus* sp. AW02J18 ISP (chain A) with the catalytic mutant *B. clausii* ISP (PDB ID: 2X8J, chain A) gave an RMSD of 0.67 Å across 282 atom pairs in an improved fit where far‐apart residues are removed (across all 292 atom pairs of residues in the alignment: 1.21 Å), confirming that they have the same overall fold (Figure 5B). Superpositioning showed that catalytic triad residues are structurally conserved, although distances are slightly different in each monomer. Two distinct conformations were modelled in each monomer due to poor electron density: Monomer A, residues 248‐252 (including the catalytic triad residue Ser251) and Monomer B, residues 16‐20 (including parts of the pro‐peptide). In monomer A the distances between Ser251Oγ and His86Nɛ2 is 3.20 and 3.58 Å, respectively, whereas the corresponding distance for monomer B measures to 3.82 Å (Figure 5C). Superpositioning with the structure representing the active state of *B. clausii* ISP (PDB ID: 2XRM) has a shorter distance, although only estimated, as both *B. clausii* structures are Ser251Ala mutants. One surface loop (residues 97‐104) is different, probably reflecting an insertion in the *Planococcus* sp. AW02J18 ISP (Supporting Information Figure S1). Although the side‐chains of some residues in this loop (residues Asp100, Glu101, and Glu102) are visible only at low contour levels, a sodium ion in each monomer was putatively identified and modeled in electron density as for the *B. clausii* ISP structures (PDB IDs: 2XRM and 2X8J).

The 2 loop regions that are disordered (residues 184‐191 and 217‐223) in *Planococcus* sp. AW02J18 ISP are ordered in the structure that simulates the active state of the *B. clausii* ISP. Residues from both loops are contributors in the coordination of a calcium ion, in *B. clausii* ISP, these are: Asp186 (side‐chain; SC), Arg188 (mainchain; MC), Thr191 (MC), Glu193 (SC), and Thr221 (SC). In *Planococcus* sp. AW02J18 ISP the residues contributing with specific side‐chain contacts to the calcium ion are conserved, while 1 of the 2 unspecific main chain contacts are not conserved (Supporting Information Figure S1).

### 3.5 Mutations in the LIPY/F motif of the pro‐peptide relieve inhibition

**Figure 6:**
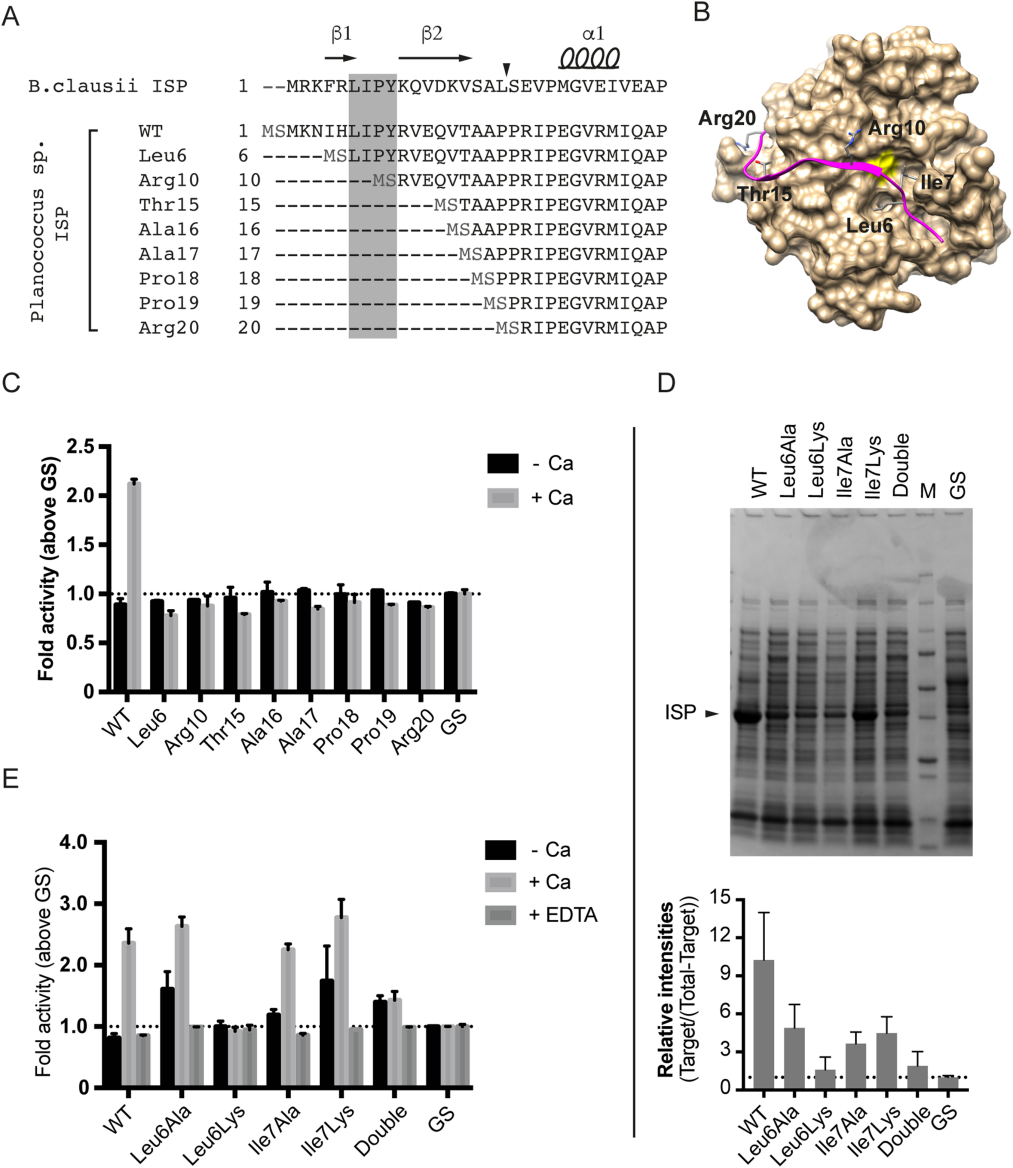
Engineering ISP at the N‐termini. A, Alignment of the N‐terminal region of ISP and the various truncated versions (indicated by starting residue given in 3‐letter ambiguity codes and their sequential numbers). A grey box indicates the LIPY/F‐motif. Beta‐strands (β), alpha helix (α), and arrow that points to the site of maturation refers to information from *B. clausii* ISP (PDB ID: 2X8J). Residues in light grey (MS) are added to the recombinant enzymes. B, Solvent‐accessible surface of *Planococcus* sp. AW02J18 ISP is shown (tan, chain A) with the catalytic serine residue in yellow. The pro‐peptide (residues 3‐20) is shown in magenta, with the Leu6 and Ile7 residues of the LIPY/F motif colored by atom. Additionally, defining residues used in the truncation experiment are indicated. C, Cleared lysates containing wild‐type ISP‐His or truncated versions (p12 constructs), were screened for activity against BODIPY‐FL‐casein in the absence and presence of 1 m*M* CaCl_2_ (+Ca) for 1 h at 37 °C. Fluorescence was normalized to optical density of expression cultures, to account for any growth effects. Lysates with empty vectors (GS) was used as background, and samples were calculated as fold above control. Error bars represent standard deviation between parallels in 2 experiments. D, A representative SDS‐PAGE analysis of cleared lysates containing wild‐type ISP‐His (WT) or mutant versions (double, both Leu6 and Ile7 mutated to Ala). M, BioRad's Precision Plus Protein™ Dual Color Standard; and GS, extracts with empty vector. Arrow points to the recombinant ISP variants. The lower panel shows intensities of target ISP proteins relative to the target corrected total lane intensity. Intensity data arise from 2 independent experiments. E, Cleared lysates from D analyzed as in C, in absence or presence of 1 m*M* CaCl_2_ (Ca) or with 1 m*M* EDTA [Color figure can be viewed at http://wileyonlinelibrary.com]

Removal of the first 18 residues of *B. clausii* ISP by calcium treatment or by truncation released an ISP enzyme in an active conformation.^5^ The proteolytic site for cleavage is however not conserved among ISPs (Supporting Information Figure S1). As calcium seemed to improve activity (Figure 2B), but also further process the Asn3‐ISP (Figure 2D), we aimed at identifying the second processing site for maturation. Despite repeated efforts, MS and N‐terminal sequencing of various protein species isolated from SDS‐PAGE gels did not reveal other processing than the removal of the 2 first residues (Supporting Information Figure S3 and Table S4). As an alternative approach, we designed various constructs where the N‐terminal region of the *Planococcus* sp. AW02J18 ISP was truncated (Figure 6A). To design a close mimic of the N‐terminus of native and processed enzyme, a p12‐based construct was chosen (ISP‐His, 38 kDa). This mimicked the full‐length ISP sequence and respective truncation mutants with C‐terminal His‐tags albeit with 2 artificial residues at the N‐terminus of recombinant enzyme (MS, Figure 6A). A Leu6 truncation construct was designed to remove the first 5 residues, not affecting the LIPY‐sequence, to assay potential detrimental effects of removal of the β1‐strand of the antiparallel β‐sheet required for structural stability (Figure 6B). An Arg10 truncation construct (that is, starting at Arg10) was designed to remove the LIPY‐sequence from the native N‐terminus, to release auto‐inhibition induced by the motif. The Thr15‐Arg20 truncations were designed to truncate the pro‐peptide in search for an active enzyme that would mimic the processed *B. clausii* ISP. Truncations beyond Arg20 were considered to be destructive as these were anticipated to interfere with secondary structure elements in the core of the catalytic domain according to the *B. clausii* ISP structures.^5^, ^6^ Positions of ISP truncations are summarized in Figure 6. None of the truncations were expected to impair the high affinity metal‐binding site or dimerization, as previous reports have identified the binding site and the dimer interface in other distant regions of the protein.^27^ According to SDS‐PAGE analysis recombinant enzymes were either not obtained or below our detection limits (Supporting Information Figure S6). Growth of *E. coli* was not affected by recombinant expression, suggesting that active enzymes, if present, were not lost due to cell death. In case the recombinant enzymes were present at undetectable levels, the truncated enzymes were assessed in an activity assay, but found not to present activity (Figure 6C).

The LIPY/F‐motif (residues 6‐9 in *Planococcus* sp. AW02J18 ISP) is conserved in pro‐peptides of ISPs (Supporting Information Figure S1). In *B. clausii* ISP the LIPY‐sequence is involved in binding the hydrophobic pocket at the active site, wherein Pro holds a critical position in displacing the scissile bond between Ile and Pro out of reach of the active site serine.^6^ According to structural data on *Planococcus* sp. AW02J18 ISP (Figure 5B) and *B. clausii* ISP^6^ the LIPY‐sequence is involved in binding the active site, potentially having critical roles in inhibiting auto‐proteolysis or cleavage of exogenous peptides. To investigate whether the LIPY/F‐motif is required for inhibition, we designed point mutations in the motif by targeting the side chains of Leu6 and Ile7, which are protruding into the hydrophobic pocket. We designed Ala and Lys mutations at both sites and a double alanine mutant (substituting both positions with Ala). According to SDS‐PAGE analysis, the Leu6Ala, and both Ile single mutants were successfully expressed, but gave lower yields than wild‐type ISP (Figure 6D). Expression levels for the Leu6Lys single mutant and the double mutant were low, if any, and variation occurred in independent experiments. The ratio of soluble protein to expressed protein was generally higher for the mutants than for wild‐type ISP (data not shown). Cleared lysates containing the wild‐type ISP and mutants were assessed in an *in vitro* BODIPY‐casein assay and compared to extracts from strains carrying the empty vector (Figure 6E). As expected, the wild‐type ISP was found to be active upon calcium treatment as determined from an increase in fluorescent signal. Upon calcium addition, the Leu6Ala, and both Ile mutants showed a similar response, but mutants showed a higher than baseline level of activity even in the absence of calcium. No activity was detected for the Leu6Lys mutant, probably because it was not expressed. The double mutant was however found to be active, despite the low expression levels. The activity of the double mutant was similar both in absence and presence of calcium, albeit low. In all cases, EDTA prevented activity, likely by chelating calcium at 1 or several binding sites.

## 4 DISCUSSION

An ISP from *Planococcus* sp. AW02J18 is herein characterized in terms of its catalytic activity, stability and structure. For recombinant expression, we explored the utility of N‐terminal His, His‐SUMO, or His‐MBP fusion tags to promote soluble expression of ISP, as previous data have shown that N‐terminal tags can be used for both intracellular^1^ and extracellular serine proteases.^32^ Expression trials showed that all fusion constructs were soluble (Figure 1). The ISP was active in the presence of calcium (Figure 2). The assumption that ISP requires pro‐peptide processing for activation, for example, as in *B. clausii* ISP, allowed exploitation of its native protease activity for intrinsic tag removal. Indeed, the construct with an N‐terminal His‐tag facilitated creation of a processed ISP without artificial tags in the presence of calcium (Figure 2 and Supporting Information Figure S3).

The ISP operates at moderate temperatures, with optimal conditions at 45 °C (Figure 3), and unfolds at about 60 °C (Figure 4). The organism of which this ISP originates, *Planococcus* sp. AW02J18, was isolated from a marine habitat, and is known to thrive at cold to moderate temperatures (data not shown). Although some ISPs are active at neutral pH,^7^ *Planococcus* sp. AW02J18 ISP, like the majority of ISPs,^2^, ^44^, ^45^, ^46^ has optimal activity at alkaline pH (Figure 3). So far, 1 ISP has been structurally characterized, namely the ISP from *B. clausii*. This study provides structural information on a second unique ISP that originates from a phylogenetically and physiologically distinct genus.^47^ The ISP crystallized mostly at acidic pH (Supporting Information Table S3), and calcium was not found in any of the crystals. The lack of activity and low processing below pH 7.0 (Figures 2 and 3) may partly explain why structures are in the inactive conformation. Whether lack of crystals at conditions above pH 7.0 is caused by degradation or because the active state does not promote crystal growth is impossible to say. Processing is not induced by pH shift alone (Figure 2D), but requires calcium. Both ISPs were found to crystallize in a dimeric state; thus, dimerization appears to be a generic feature of ISPs. According to size exclusion chromatography, the presence of calcium the Asn3‐ISP lead to a mixed population of quaternary structures corresponding to approximately 2.5 and 3.7 monomers per oligomer (Supporting Information Figure S5). Whereas the dimeric form is confirmed in the crystal, the oligomeric state in solution was inconclusive. It appeared however that the presence of calcium induced 2 new states compared to the calcium‐depleted enzyme solutions. In accordance with earlier observations, calcium depletion may lead to a more compact structure (here represented by the shift from 3.7 to 3.3 monomers per oligomer state). The presence of calcium may induce autoproteolysis, thereby reducing the apparent molecular weight (here represented by the shift from 3.3 to 2.5 monomers per oligomers). The higher molecular weight induced by the presence of calcium (here represented by the 3.7 monomer per oligomer state) may arise from a less compact structure or even from aggregation. The 2 monomers contained regions of poor electron density in proximity to each other. These are most likely partially flexible regions as a consequence of the structural reorganization caused by the insertion of the pro‐peptide in the substrate‐binding region. The C‐terminal 20 residues were not defined in electron density, while in 2 different crystal forms representing structures of ISP from *B. clausii* (PDB ID: 2X8J and 2WWT), these residues are stabilized through interactions with symmetry mates. According to sequence alignments, the C‐terminal region is not conserved (Supporting Information Figure S1), but the reason for this region being flexible in the structure of *Planococcus* sp. AW02J18 ISP is not clear. Ultimately, the requirement and role of the C‐terminal residues in folding and dimerization of ISPs remains unclear.

From studies of *B. clausii* ISP, divalent metal ions, possibly calcium, bind close to the S1 pocket.^5^, ^6^ In the crystals of *Planococcus* sp. AW02J18 ISP, calcium was not identified at any of the metal binding site. Two loop regions were not defined in the electron density of ISP, which is also the case for the *B. clausii* ISPs containing the intact pro‐peptide (PDB IDs: 2X8J and 2WWT). These loop regions are however ordered in the *B. clausii* ISP structure that simulates the active conformation of the enzyme, albeit with a catalytic mutation (PDB ID: 2XRM). Residues from both loops contribute to the coordination of a calcium ion, and these residues are conserved in aligned sequences (Supporting Information Figure S1). This could indicate a specific role of calcium in the transition from inactive to active enzyme, not only for the *B. clausii* ISP, but also for other ISPs. Asn3‐ISP from *Planococcus* sp. AW02J18 was active in presence of calcium, but susceptible to self‐degradation (Figure 2). The fact that ISPs were not active without exogenous addition of calcium suggests that available metal binding sites were not occupied after production. Due to conservation of calcium‐coordinating residues (Supporting Information Figure S1), and the need for high EDTA concentrations to inhibit activity (Figure 2C), low affinity for calcium is likely not the case. DSC results suggest that additional calcium is only slightly stabilizing, and tightly bound calcium (removable with EDTA) is not essential for overall stability (Figure 4). DSC showed however that calcium does have a minor stabilizing effect; thus suggesting that the added calcium in our assays contribute to minor structural rearrangements.

It is likely that there are structural rearrangements, such as pro‐peptide flip‐out or removal, in order for the 2 loops to order and coordinate calcium. The IP residues of the LIPY/F motif in the pro‐peptide are spatially close to residues in 1 of the loops that need to be reoriented upon calcium binding. The 2 residues form hydrophobic interactions to the side‐chain of Phe195 in our inactive structure and probably hinder this reorienting into the active conformation (this side‐chain appears to be shifted almost 15 Å in the active state).

It is likely that the pro‐peptide in the ISP from *Planococcus* sp. AW02J18 is removed, in analogy to several *Bacillus* ISPs.^5^, ^7^ From the available structures of ISPs with intact pro‐peptides (PDB ID: 6F9M, 2X8J, 2WWT, 2WVT, whereof 2 are shown in Figure 5B) and the sequence alignment (Supporting Information Figure S1), we found that 2 short beta‐strands in the pro‐peptide are likely structurally conserved. The secondary structure elements are stabilized by main chain interactions, which are sequence independent. A unique feature of the *Planococcus* sp. AW02J18 ISP that is not found in homologous ISPs is the presence of the 2 consecutive proline residues in the transition between the pro‐peptide and the catalytic domain (Supporting Information Figure S1). The removal of the ISP pro‐peptide in *Planococcus* sp. AW02J18 appears to be different, and possibly involves several steps (Figure 2D). In the first step the 2 first residues of the ISP (Met1, Lys2) are removed (protein band numbered 2, Figure 2D), as identified in the crystal and by N‐terminal sequencing. Another product, which appears as the main product (around 30‐35 kDa) at pH 8.5 in presence of 10 m*M* CaCl_2_ (protein band numbered 3, Figure 2D), could possibly be functional. This product could in principle arise from processing of the C‐terminal region of the protein, too, albeit not identified in the crystal or MS analyses (Supporting Information Figure S5 and Table S4). The N‐terminal residues of this protein could not be identified. Unfortunately, MS analyses did not reveal obvious processing patterns at the N‐terminal in the protein species from the SDS‐PAGE analysis (Supporting Information Figure S5 and Table S4). This may partly be due to a lack of sequential degradation. Ultimately, we could not determine which ISP moiety that is responsible for or contribute to the activity identified in assays. A truncation experiment was conducted to trim the pro‐peptide in the hunt for processing site(s). Two artificial residues (Met‐Ser) are unavoidably added to the N‐terminal end of these truncation constructs, which arise from fusion of the *isp* gene fragment to the start codon and the ligation seam added during sub‐cloning (Figure 6A), and their negative interference on protein stability cannot be ruled out. Sequence analysis of *Planococcus* sp. AW02J18 ISP, reveals that it contains 2 prolines in the transition from the pro‐peptide to the catalytic domain (Supporting Information Figure S1). Whereas Pro at the P2 site is likely accepted, Pro at the P1 is highly unlikely due to the preference of hydrophobic residues at the S1 site.^27^ Multiple prolines are normally not present in sites for autoproteolysis by serine proteases,^48^ and the prolines may instead serve a structural role.^49^ This does not however rule out that other proteases, for example proline‐specific endopeptidases, could process and remove the pro‐peptide in native conditions, or that processing site(s) are in other regions that were not included in this study.

**Figure 7:**
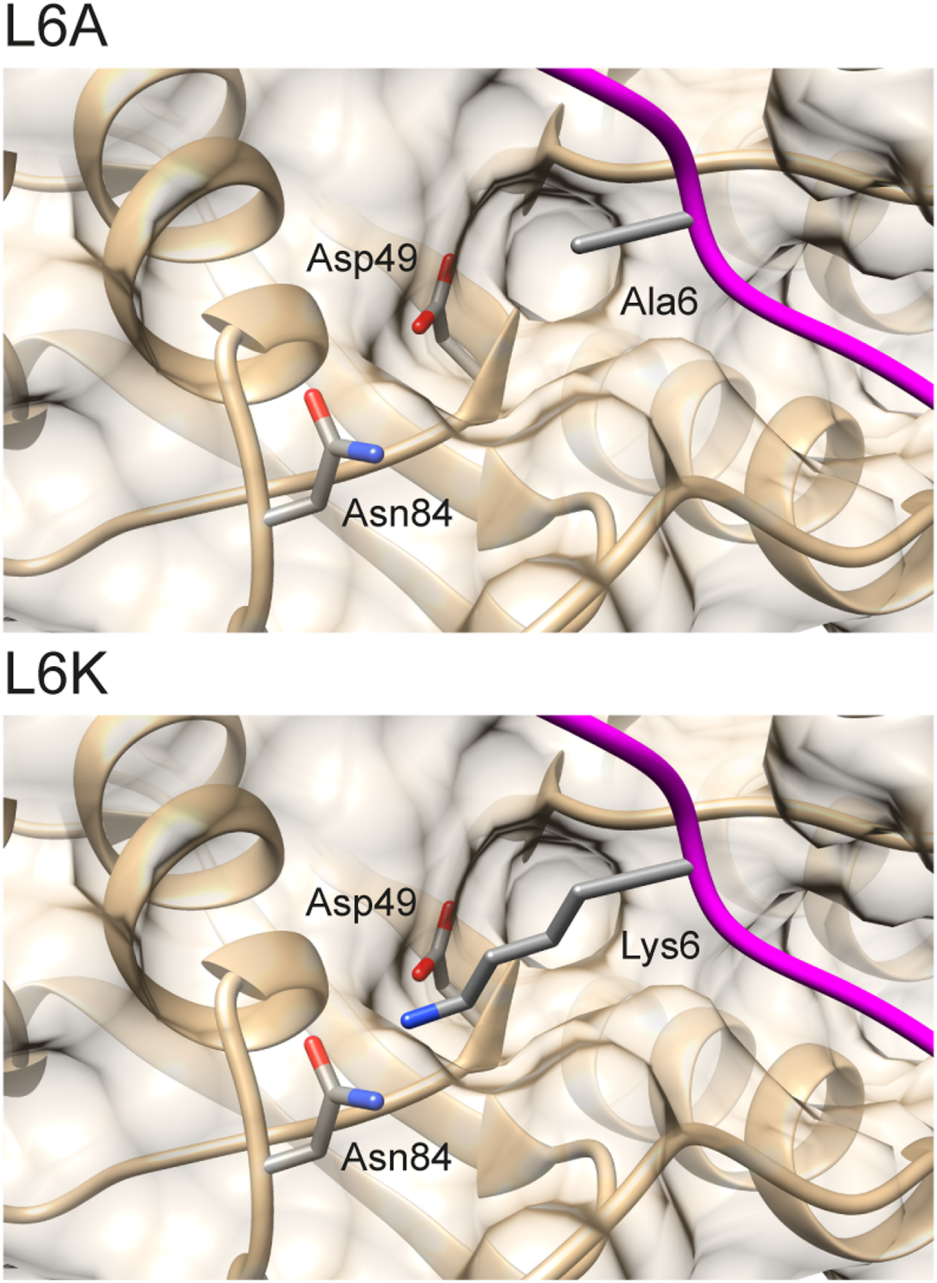
Mutation of Leu6 in the LIPY/F motif. Solvent‐accessible surface of *Planococcus* sp. AW02J18 ISP (B‐chain) is shown in opaque tan. The backbone and secondary elements is represented in ribbons, expect for the pro‐peptide which is shown in magenta. The side chains of mutants (Ala6, Lys6), Asp49 and Asn84 are colored by atomic elements [Color figure can be viewed at http://wileyonlinelibrary.com]

Although it has been found that the pro‐peptide of *B. clausii* ISP has a role in inhibition, the contribution of the conserved residues within the LIPY/F‐motif has not been studied in detail. Due to the fact that Leu and Ile are conserved in the motif, and that the ISPs likely prefer hydrophobic amino acids at the S2 and S4 sites,^27^ we studied point mutations of Leu6 and Ile7 in *Planococcus* sp. AW02J18 ISP. Data form 3 of the 4 single point mutations, which resulted in increased activity ‐ even in the absence of excessive calcium, indicate that Leu6 and Ile7 have substantial roles in inhibition and support the involvement of calcium during activation. A closer inspection of the structural context suggests that substitution of Leu6 with Ala likely reduced the hydrophobic interaction to the active site, and thus relieves the inhibition (Figure 7). The substitution to lysine however seems to both reduce expression level (Figure 6D). It furthermore does not respond on calcium addition in the activity assay (Figure 6E). Assuming that the mutant is properly folded, inhibition could be explained by the possibility that lysine can form hydrogen bond and/or salt bridge interactions with the catalytic Asp49 and the Asn84 residues, respectively (Figure 7). Structural explanations for the Ile7 mutants were not conclusive due to their proximity to the flexible region (183‐193), but it is likely that both mutations cause reduced interactions with the pro‐peptide. We thus conclude that the pro‐peptide, with the LIPY/F motif in a central position, is involved in inhibition. Our data is in line with the proposed ISP model,^27^ suggesting that calcium binding at the active site is prevented during pro‐peptide inhibition.

## AUTHOR CONTRIBUTIONS

G.E.K.B. designed the study, designed and supervised experiments and drafted the manuscript, Ø.L. carried out expression, purification, and biochemical assays, H.A. set up crystallization trials and performed the DSC experiment, I.L. performed data collection, processed data, determined the structure and refined it, A.G.M. performed phylogenetic analysis, A.W. supervised and designed the DSC experiment. P.P. supervised mutant design and performed bioinformatic analyses. All authors were involved in revision of the manuscript and approved the final version.

## ACKNOWLEDGMENTS

We would like to thank Arne O. Smalås for sharing sequence data, Stefan Hauglid and Trine Carlsen for expert assistance on crystallization and support of staff during visit, and Hilde Eide Lien for excellent work on truncation and mutant studies. Provision of beam time at the European Synchrotron Radiation Facility (ESRF) is highly valued. The authors thank the Research Council of Norway for financial support (221568). Authors declare no conflict of interest.

## Supporting information


**Supporting Information**
