# Supplementary material for: Mutational analysis of the pro‐peptide of a marine intracellular subtilisin protease supports its role in inhibition
Source: Proteins. 2018 Sep 17;86(9):965–77. doi: 10.1002/prot.25528 (PMC6220982; doi:10.1002/prot.25528)
Supplement: Supplementary file 1 — Supporting Information [file PROT-86-965-s001.docx]

*Supplementary data*

**Mutational analysis of the pro-peptide of a marine intracellular subtilisin protease supports its role in inhibition**

Short title: Pro-domain in marine ISP

Gro E. K. Bjerga^1*^, Øivind Larsen^1^, Hasan Arsın^2^, Adele Williamson^3^, Antonio García-Moyano^1^, Ingar Leiros^3^, Pål Puntervoll^1^

^1^Uni Research, Center for applied biotechnology, Thormøhlens gate 55, 5006 Bergen, Norway

^2^University of Bergen, Department of biology, Thormøhlens gate 53, 5006 Bergen, Norway

^3^ UiT The Arctic University of Norway, Department of Chemistry, N-9037 Tromsø, Norway

**Table S1: Information on the ISP candidate**

| ORF tag | PRTUIT00110 |
| --- | --- |
| Isolate (NCBI taxonomy ID) | *Planococcus* sp. AW02J18 (1379956) |
| Isolate origin (Degrees Lat Long) | Lofoten (68.5025473N°, 015.0046585E°) |
| Isolate source (depth in meters) | Biota (135) |
| Length (aa) | 329 |
| SignalP leader sequence | No |
| Pfam domain (name) | Peptidase_S08 |
| Pfam domain (aa) | 40-311 |
| Closest MEROPS hit (ID) | MER324776 |
| Closest MEROPS organism | *Planococcus donghaensis* |
| Identity (%) | 72.0 |

**Table S2: Primers used in this study**

| Primer name | Sequence (5'-3') | Purpose |
| --- | --- | --- |
| T0034-S251A-F | CTGTCGGGTACCGCTATGGCTACGC | Mutagenesis |
| T0034-S251A-R | GCGTAGCCATAGCGGTACCCGACAG | Mutagenesis |
| T0034-L06-FX-F | ATATATGCTCTTCTAGTCTGATCCCGTATCGTGTGG | Truncation |
| T0034-R10-FX-F | ATATATGCTCTTCTAGTCGTGTGGAACAGGTTACCG | Truncation |
| T0034-T15-FX-F | ATATATGCTCTTCTAGTACCGCCGCCCCGCCGCG | Truncation |
| T0034-A16-FX-F | ATATATGCTCTTCTAGTGCCGCCCCGCCGCGTATTCC | Truncation |
| T0034-A17-FX-F | ATATATGCTCTTCTAGTGCCCCGCCGCGTATTCCG | Truncation |
| T0034-P18-FX-F | ATATATGCTCTTCTAGTCCGCCGCGTATTCCGG | Truncation |
| T0034-R20-FX-F | ATATATGCTCTTCTAGTCGTATTCCGGAAGGCGTCCG | Truncation |
| T0034-L6A-F | GAAAAACATTCATGCGATCCCGTATCG | Mutagenesis |
| T0034-L6A-R | CGATACGGGATCGCATGAATGTTTTTC | Mutagenesis |
| T0034-I7A-F | CATTCATCTGGCCCCGTATCGTG | Mutagenesis |
| T0034-I7A-R | CACGATACGGGGCCAGATGAATG | Mutagenesis |
| T0034-L6AI7A-F | ACATTCATGCGGCCCCGTATCG | Mutagenesis |
| T0034-L6AI7A-R | CGATACGGGGCCGCATGAATGT | Mutagenesis |
| T0034-L6K-F | GAAAAACATTCATAAGATCCCGTATCG | Mutagenesis |
| T0034-L6K-R | CGATACGGGATCTTATGAATGTTTTTC | Mutagenesis |
| T0034-I7K-F | CATTCATCTGAAACCGTATCGTG | Mutagenesis |
| T0034-I7K-R | CACGATACGGTTTCAGATGAATG | Mutagenesis |

**Table S3: Crystallization conditions**

| Crystal | Crystallization condition | Image |
| --- | --- | --- |
| 1 | 0.1 M Sodium Citrate pH 5, 0.51 % Ethylene glycol, 20 % PEG 4000 | 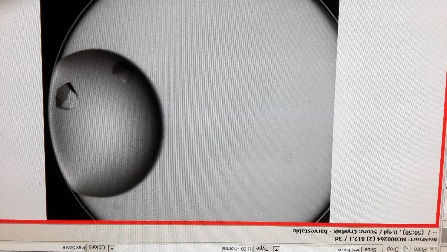 |
| 2 | 0.17 M Na-K-Phosphate, 27 % PEG MME 2000 | 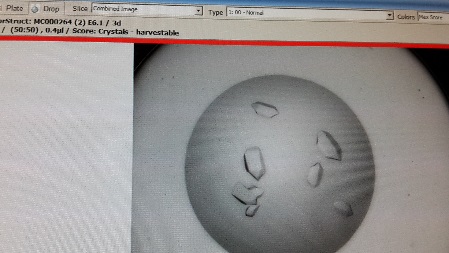 |
| 3 | 0.1 M Sodium Citrate pH 5.5, 24 % PEG 6000 | 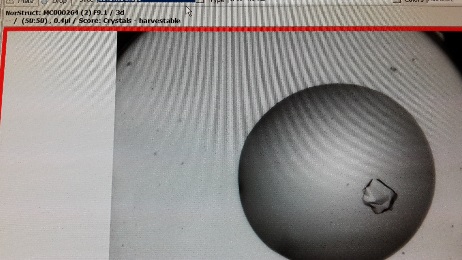 |
| 4 | 0.1 M Sodium Citrate pH 4, 0.25 M Ammonium Acetate, 22 % PEG 1500 | 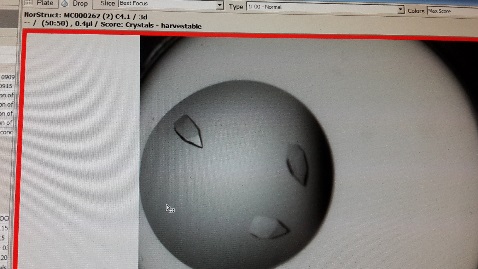 |
| 5 | 0.09 M Ammonium Formate, 0.1 M Sodium Citrate, 20 % PEG 1500 | 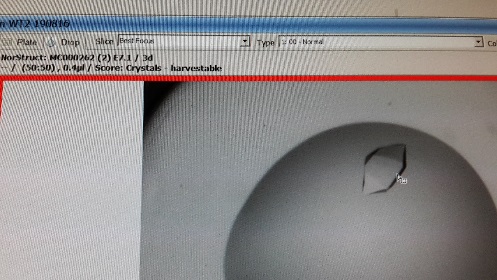 |
| 6 | 0.1 M Phosphate Citrate Buffer pH 4.5, 0.2 M LiSO4, 20 % PEG 1500 | 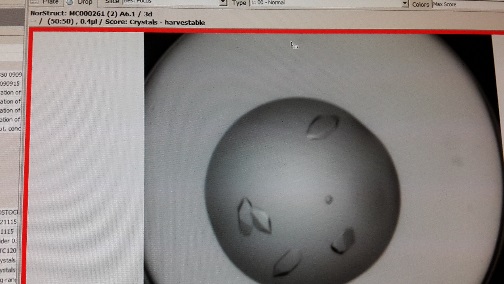 |

**Table S4: Mass spectrometry analysis.** Refer to Figure S3 for sample information.

| **AA Sequence** | **First AA** | **Last AA** | **Modifications** | **Sample #1** | **Sample #2** | **Sample #3** | **Sample #4** | **Sample #5** |
| --- | --- | --- | --- | --- | --- | --- | --- | --- |
| [K].NIHLIPYRVEQVTAAPPR.[I] | 24 | 41 |  | 6 | 17 | 7 | 18 | 3 |
| [K].NIHLIPYR.[V] | 24 | 31 |  | 102 | 58 | 11 | 33 | 8 |
| [R].VEQVTAAPPR.[I] | 32 | 41 |  | 114 | 108 | 6 | 69 | 4 |
| [R].IPEGVR.[M] | 42 | 47 |  | 1 |  |  |  |  |
| [R].MIQAPELWESAEHGK.[G] | 48 | 62 | 1xOxidation [M1] | 92 | 49 | 66 | 36 | 12 |
| [R].MIQAPELWESAEHGK.[G] | 48 | 62 |  | 17 | 19 | 18 | 13 | 4 |
| [R].MIQAPELWESAEHGKGNVVAVLDTGCQTDHPDLTAR.[I] | 48 | 83 | 1xCarbamidomethyl [C26] |  | 1 | 1 |  |  |
| [K].GNVVAVLDTGCQTDHPDLTAR.[I] | 63 | 83 | 1xCarbamidomethyl [C11] | 101 | 139 | 159 | 60 | 3 |
| [R].NFTHDDGGDPERFEDYNGHGTHVAGTVAASLR.[D] | 89 | 120 |  | 23 | 8 | 14 | 4 |  |
| [R].NFTHDDGGDPER.[F] | 89 | 100 |  | 35 | 13 | 50 | 29 | 4 |
| [R].FEDYNGHGTHVAGTVAASLR.[D] | 101 | 120 |  | 8 | 4 | 8 | 8 | 2 |
| [R].FEDYNGHGTHVAGTVAASLRDEEGVVGVAPLADLLVVK.[V] | 101 | 138 |  | 9 | 9 | 13 |  |  |
| [R].DEEGVVGVAPLADLLVVK.[V] | 121 | 138 |  | 74 | 74 | 66 | 38 | 4 |
| [R].DEEGVVGVAPLADLLVVKVLDK.[E] | 121 | 142 |  | 10 | 1 | 2 | 4 |  |
| [K].VLDKEGSGSYEGIIAGIHYAIDWR.[G] | 139 | 162 |  | 106 | 113 | 106 | 8 | 6 |
| [K].VLDKEGSGSYEGIIAGIHYAIDWRGPEGQK.[T] | 139 | 168 |  | 2 | 5 | 7 |  |  |
| [K].EGSGSYEGIIAGIHYAIDWR.[G] | 143 | 162 |  | 80 | 78 | 88 | 2 | 2 |
| [K].EGSGSYEGIIAGIHYAIDWRGPEGQK.[T] | 143 | 168 |  | 3 | 4 | 3 |  |  |
| [R].GPEGQKTTVISMSLGGPEDHPELYEAVK.[R] | 163 | 190 |  | 4 |  |  |  |  |
| [R].GPEGQKTTVISMSLGGPEDHPELYEAVK.[R] | 163 | 190 | 1xOxidation [M12] | 2 |  |  |  |  |
| [R].GPEGQKTTVISMSLGGPEDHPELYEAVKR.[A] | 163 | 191 |  | 2 |  |  |  |  |
| [R].GPEGQKTTVISMSLGGPEDHPELYEAVKR.[A] | 163 | 191 | 1xOxidation [M12] | 1 |  |  |  |  |
| [K].TTVISMSLGGPEDHPELYEAVKR.[A] | 169 | 191 | 1xOxidation [M6] | 64 | 32 | 60 | 20 | 4 |
| [K].TTVISMSLGGPEDHPELYEAVK.[R] | 169 | 190 | 1xOxidation [M6] | 34 | 21 | 30 | 15 | 2 |
| [K].TTVISMSLGGPEDHPELYEAVK.[R] | 169 | 190 |  | 44 | 28 | 30 | 17 | 2 |
| [K].TTVISMSLGGPEDHPELYEAVKR.[A] | 169 | 191 |  | 62 | 43 | 53 | 5 | 4 |
| [K].RAVDAGIPVICAAGNEGDDAHDTDEFAYPGAYGEVIQVGAVDFDR.[R] | 191 | 235 | 1xCarbamidomethyl [C11] | 2 |  |  |  |  |
| [K].RAVDAGIPVICAAGNEGDDAHDTDEFAYPGAYGEVIQVGAVDFDRR.[I] | 191 | 236 | 1xCarbamidomethyl [C11] | 2 | 3 | 2 |  |  |
| [R].AVDAGIPVICAAGNEGDDAHDTDEFAYPGAYGEVIQVGAVDFDR.[R] | 192 | 235 | 1xCarbamidomethyl [C10] | 10 | 5 | 7 |  |  |
| [R].AVDAGIPVICAAGNEGDDAHDTDEFAYPGAYGEVIQVGAVDFDRR.[I] | 192 | 236 | 1xCarbamidomethyl [C10] | 9 | 12 | 17 | 2 |  |
| [R].RIAPFSNTNNEIDLVAPGINIYSTYLEGK.[Y] | 236 | 264 |  | 2 | 6 | 5 |  |  |
| [R].IAPFSNTNNEIDLVAPGINIYSTYLEGK.[Y] | 237 | 264 |  | 29 | 31 | 24 | 2 | 2 |
| [K].YASLSGTSMATPHVSGALALIR.[N] | 265 | 286 |  | 61 | 64 | 58 | 4 | 51 |
| [K].YASLSGTSMATPHVSGALALIR.[N] | 265 | 286 | 1xOxidation [M9] | 105 | 99 | 111 | 6 | 113 |
| [K].YASLSGTSMATPHVSGALALIRNISER.[E] | 265 | 291 | 1xOxidation [M9] |  |  |  |  | 4 |
| [R].NISEREFDRELTEAELYAQLVR.[R] | 287 | 308 |  |  | 4 | 6 |  | 7 |
| [R].EFDRELTEAELYAQLVR.[R] | 292 | 308 |  | 101 | 89 | 109 | 10 | 91 |
| [R].EFDRELTEAELYAQLVRR.[T] | 292 | 309 |  | 1 |  | 2 |  | 4 |
| [R].ELTEAELYAQLVR.[R] | 296 | 308 |  | 116 | 110 | 114 | 2 | 79 |
| [R].ELTEAELYAQLVRR.[T] | 296 | 309 |  | 1 |  |  |  | 2 |
| [R].RTIPLGYPK.[T] | 309 | 317 |  | 15 | 10 | 22 | 4 | 11 |
| [R].TIPLGYPKTAEGNGLLALDILNK.[F] | 310 | 332 |  | 2 |  |  |  |  |
| [R].TIPLGYPK.[T] | 310 | 317 |  | 2 | 4 | 4 | 2 | 2 |
| [K].TAEGNGLLALDILNK.[F] | 318 | 332 |  | 32 | 8 | 6 | 2 | 8 |
| [K].TAEGNGLLALDILNKFEQLFK.[I] | 318 | 338 |  | 11 | 4 | 2 | 2 | 2 |
| [K].FEQLFKILSNSYANGLDR.[A] | 333 | 350 |  | 2 |  |  |  |  |
| [K].FEQLFK.[I] | 333 | 338 |  | 88 |  | 2 | 2 |  |
| [K].ILSNSYANGLDR.[A] | 339 | 350 |  | 4 |  |  |  |  |
| [K].ILSNSYANGLDRA.[-] | 339 | 351 |  | 2 |  |  |  |  |

**
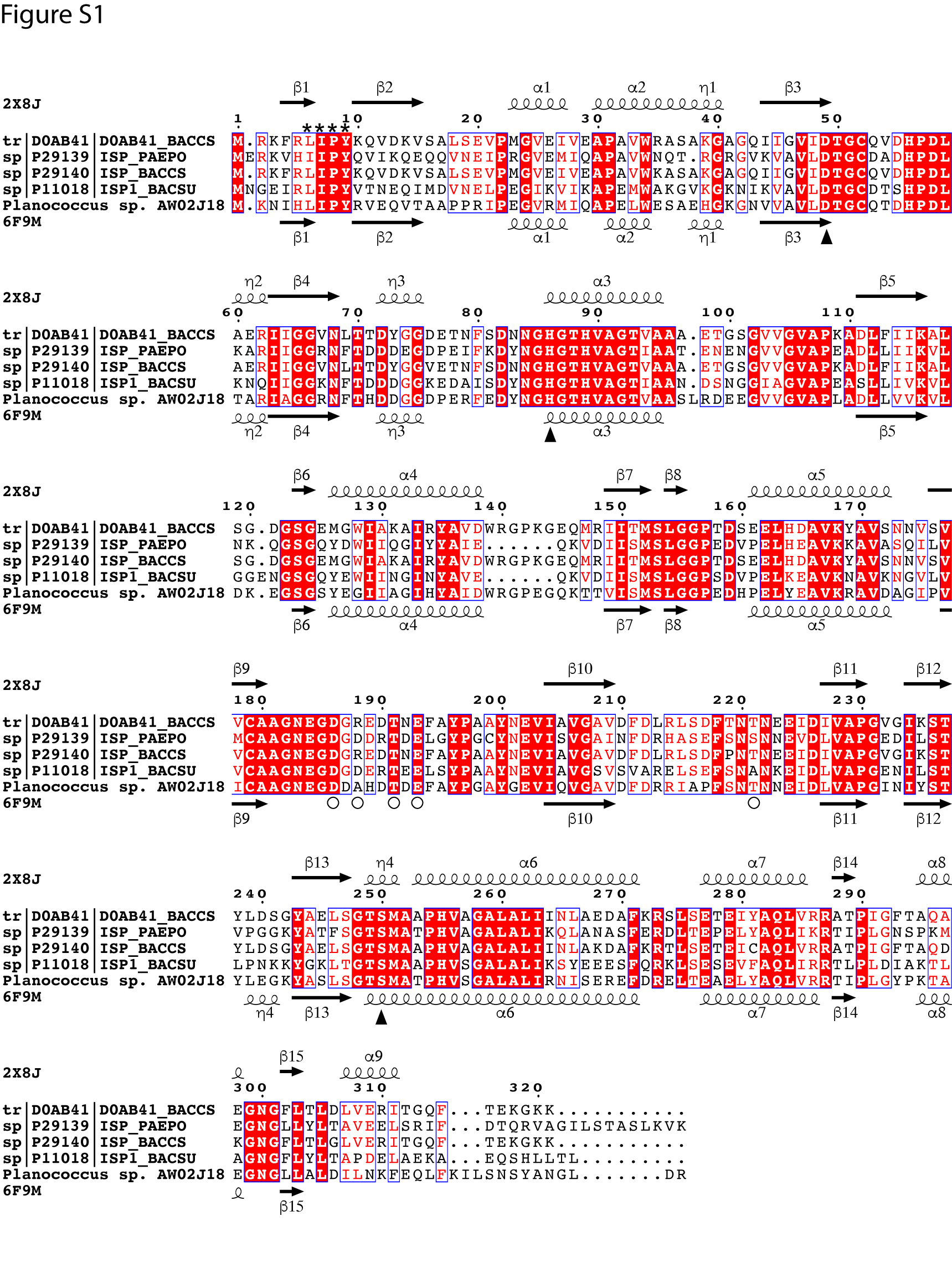
**

**Figure S1. Sequence alignment of *Planococcus* sp. AW02J18** **ISP and homologs.** Alignment of ISPs from *B. clausii* (UniProt ID: D0AB41, P29140), *Paenibacillus polymyxa* (UniProt ID: P29139), *B. subtilis* sp. 168 (UniProt ID: P11018) and *Planococcus* sp. AW02J18. Secondary structure annotations are retrieved from *B. clausii* (PDB ID: 2X8J, chain A) and *Planococcus* sp. AW02J18 ISPs (PDB ID: 6F9M). Arrows and spirals indicate β-strands and α-helices, respectively. Asterisks point to the conserved LIPY/F motif. Conserved residues are shown in red, wherein red backgrounds indicate identical residues and red letters indicate similar residues. Positions indicated with filled triangles represent the catalytic triad (Asp49, His86, Ser251 in *Planococcus* sp. AW02J18). Aligned sequences were made with MAFFT and rendered by ESPript3.0 ^1^.

**
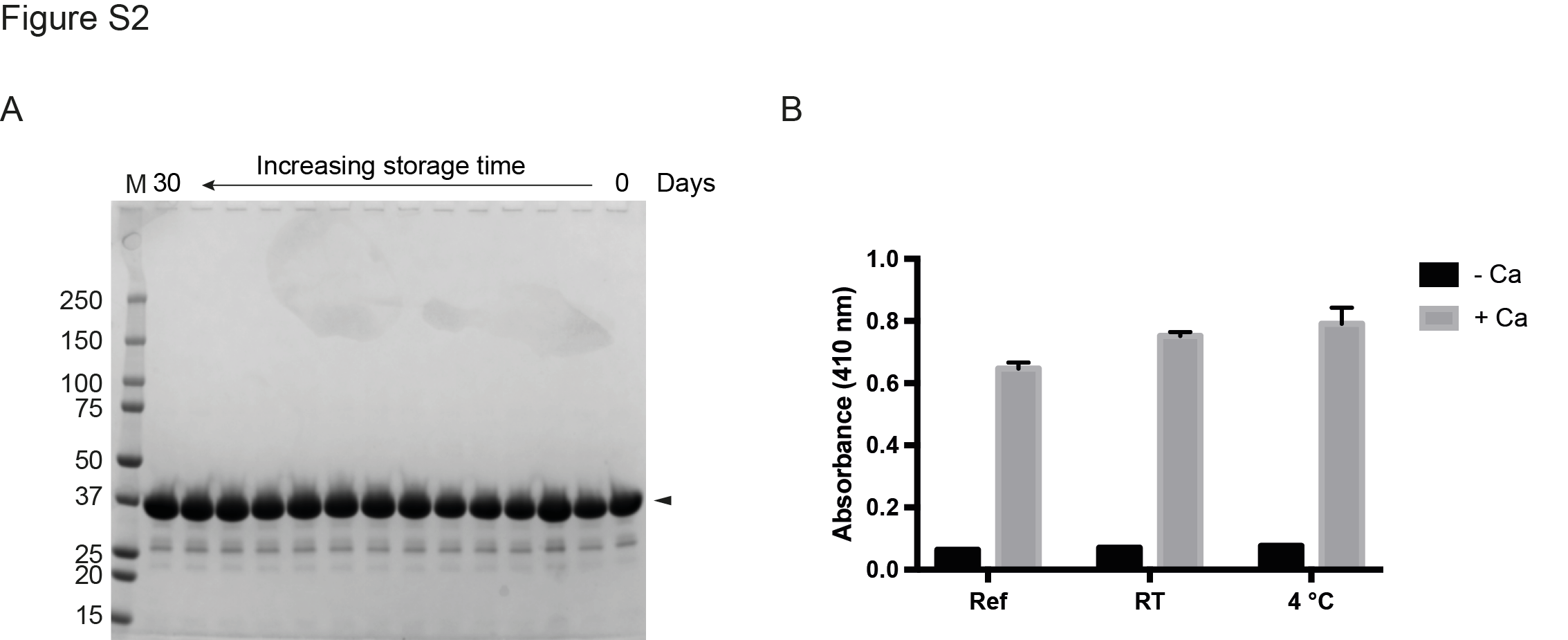
**

**Figure S2. Storage stability of Planococcus sp. AW02J18 ISP. A)** SDS-PAGE analysis of matured His-ISP. Samples were withdrawn from the frozen (-20 °C) aliquote at least every fourth day and analysed for protelysis. **B)** After 30 days of storage at ambient temperature (RT) and in the frigde (4 °C), activity was assessed on pNA-AAPF peptide in the presence (+ Ca) and absence of calcium (- Ca) and compared to a reference sample which was withdrawn from the frozen (-20 °C) batch immediately before assaying.


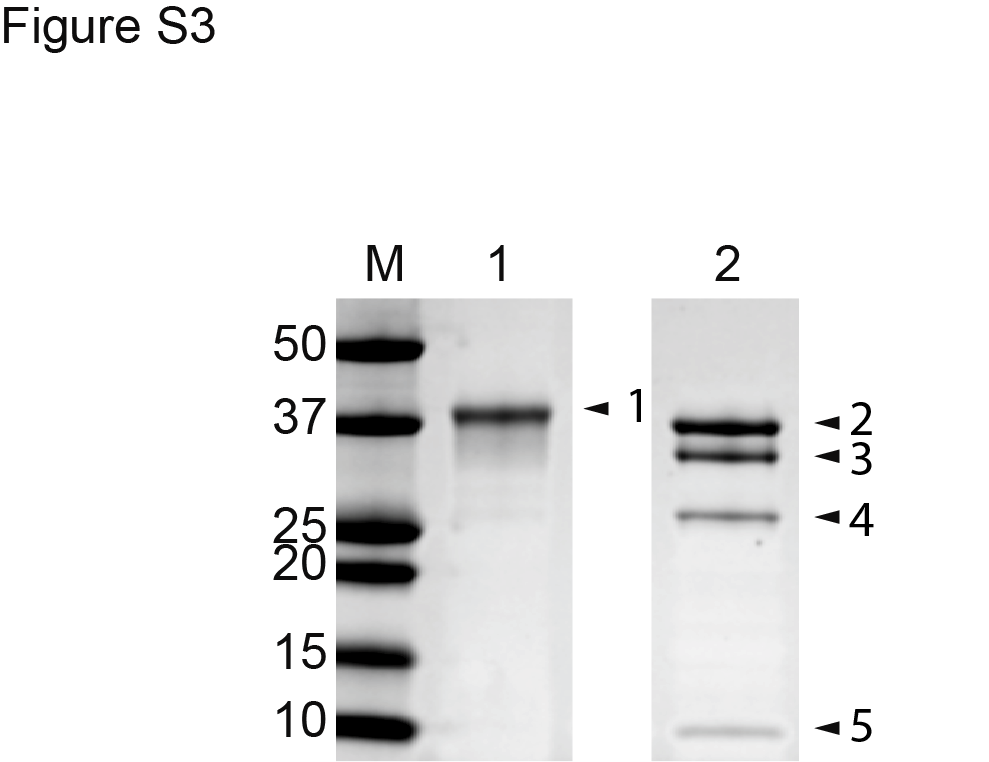


**Figure S3. ISP samples used for mass spectrometry analysis.** SDS-PAGE analysis of His-ISP (p1-construct; Lane 1) and matured versions after calcium treatment (Lane 2). Arrows point to the different protein entities, and numbers 1-5 refer to mass spectrometry samples in Table S4. M, BioRad’s Precision Plus Protein™ Dual Color Standard.

**
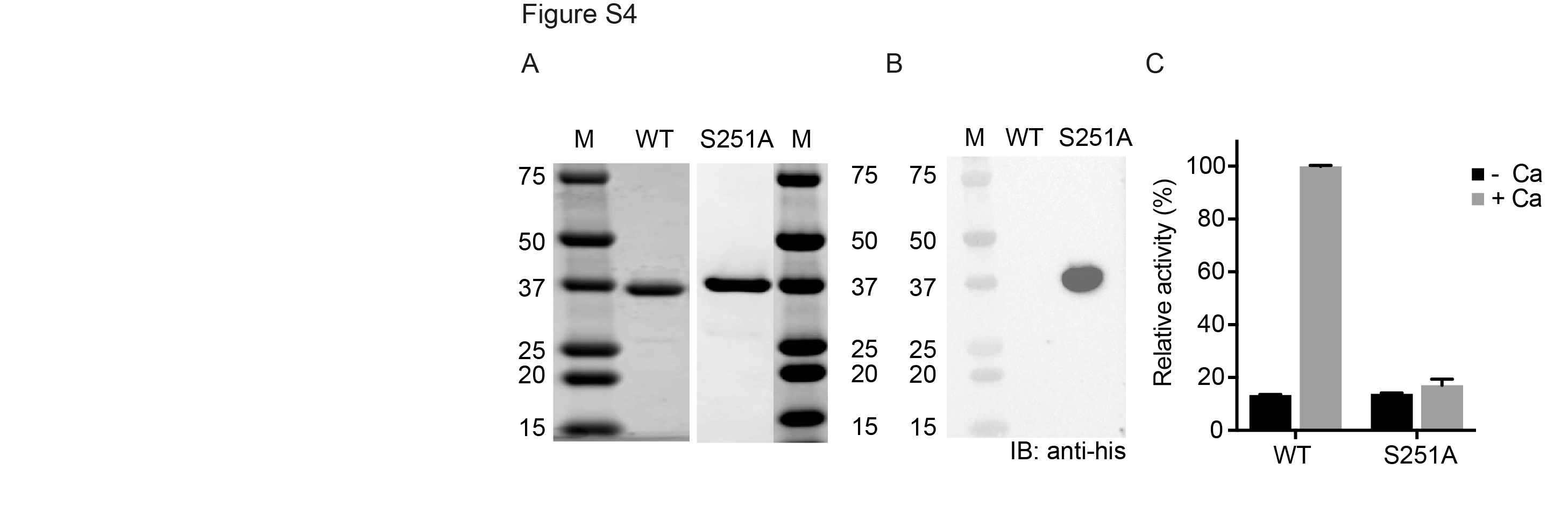
**

**Figure S4**. **Purity and activity of *Planococcus* sp. AW02J18 ISP and a catalytic mutant.** A**)** Purity of matured His-ISP from *Planococcus* sp. AW02J18 (WT, p1-construct) and the catalytic ISP-His mutant (S251A, Ser251Ala; p12-construct) after purification as analyzed by SDS-PAGE. Both proteins are >95% pure, as identified by quantitative analysis. M, BioRad’s Precision Plus Protein™ Dual Color Standard. **B)** Immunoblot analysis of the proteins in B. A standard immunoblot analysis using 1:3000 dilution of anti-His antibody was used to identify the presence of His-tags in proteins (as in A). M, as in A. **C)** The activities of matured ISP (WT) and the catalytic mutant (as in A) from crude extracts (both p12-constructs) were compared in the FITC-casein assay in the absence and presence of 1 mM CaCl_2_ to verify that the serine mutation abolishes the catalytic power.


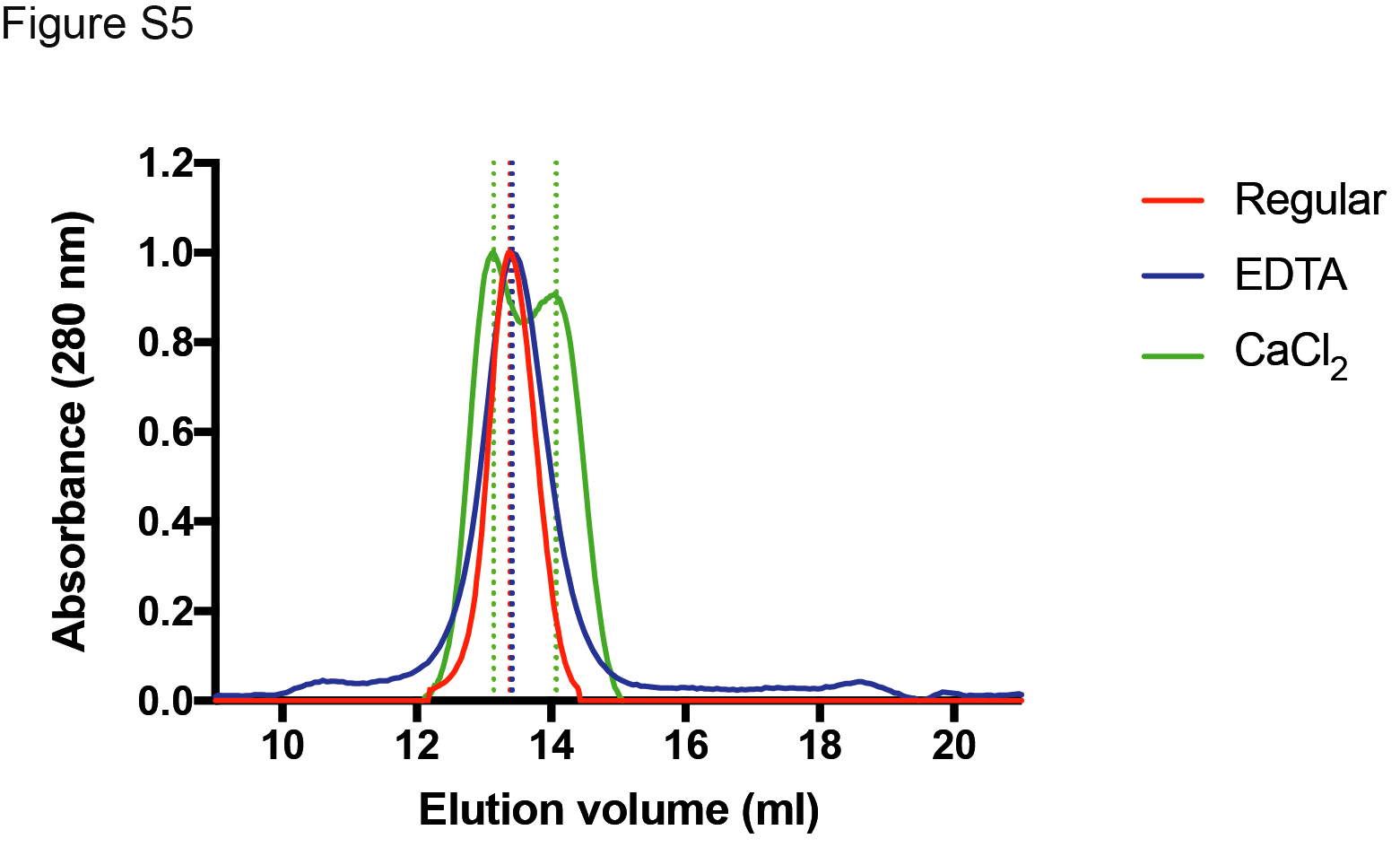


**Figure S5. Influence of calcium and EDTA on the quaternary structure of ISP.** Elution profiles from size exclusion chromatography of Asn3-ISP where calcium was removed by extensive buffer exchange (regular; red line), in presence of 1 mM EDTA (blue line) or in presence of 1 mM CaCl_2_ (green line). The vertical dashed lines indicate the peak elution volume for each sample.

**
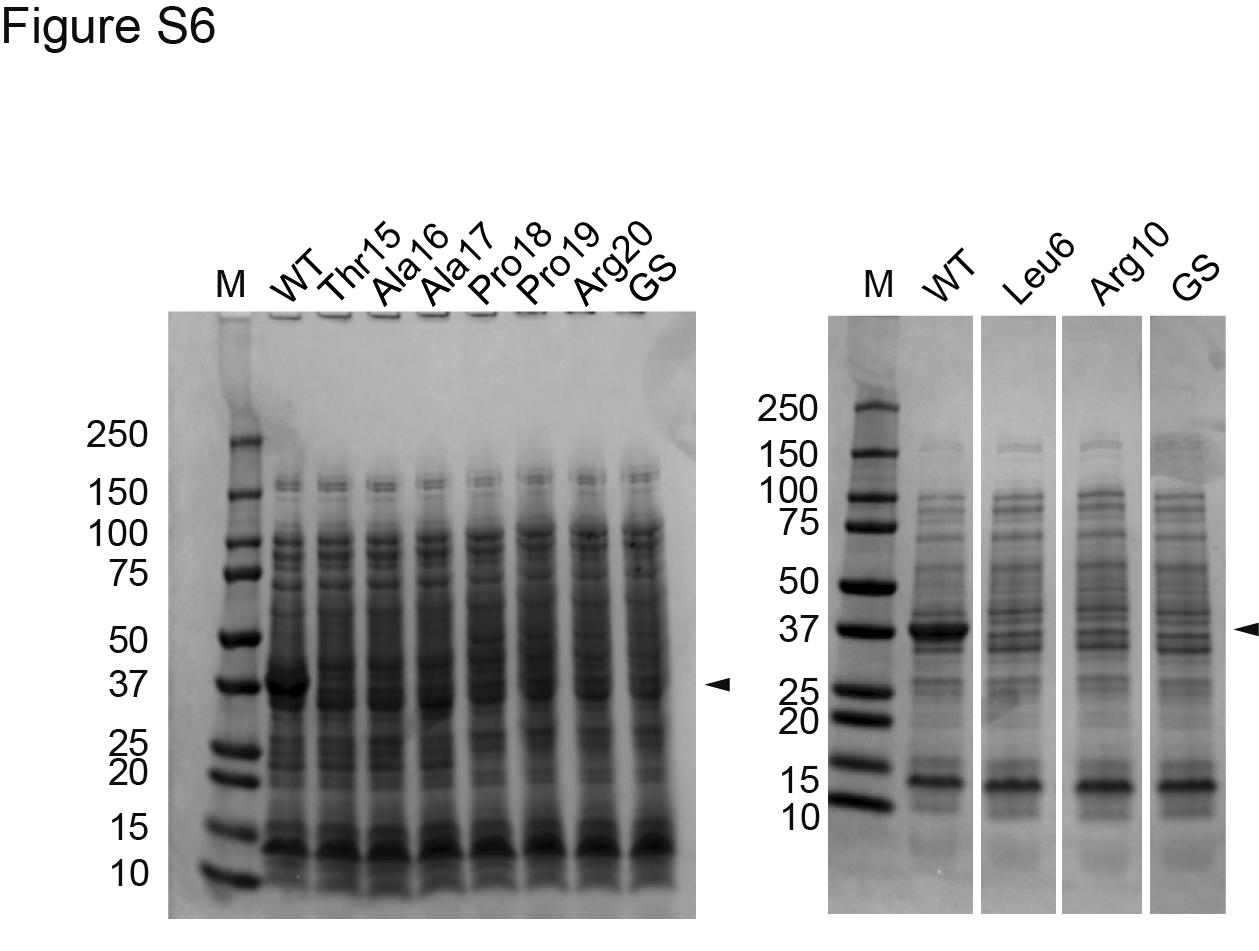
**

**Figure S6. Recombinant expression of truncated ISP versions.** SDS-PAGE analysis of cleared lysates containing truncated versions of ISP-His (WT). M, BioRad’s Precision Plus Protein™ Dual Color Standard; and GS, extracts with empty vector. Left panel shows expression of Thr15 to Arg20 truncation constructs, whereas right panel shows Leu6 and Arg10 truncation constructs. Arrows point to recombinant enzymes (WT is approx. 38 kDa).

**REFERENCE**

(1) Robert, X., and Gouet, P. (2014) Deciphering key features in protein structures with the new ENDscript server. *Nucleic Acids Res.* *42*, 320–324.
